# Dynamic Virtual Network Reconfiguration Method for Hybrid Multiple Failures Based on Weighted Relative Entropy

**DOI:** 10.3390/e20090711

**Published:** 2018-09-15

**Authors:** Yuze Su, Xiangru Meng, Qiaoyan Kang, Xiaoyang Han

**Affiliations:** College of Information and Navigation, Air Force Engineering University, Xi’an 710077, China

**Keywords:** virtual network, weighted relative entropy, reconfiguration, hybrid multiple failures, survivability

## Abstract

Network virtualization can offer more flexibility and better manageability for next generation Internet. With the increasing deployments of virtual networks in military and commercial networks, a major challenge is to ensure virtual network survivability against hybrid multiple failures. In this paper, we study the problem of recovering virtual networks affected by hybrid multiple failures in substrate networks and provide an integer linear programming formulation to solve it. We propose a heuristic algorithm to tackle the complexity of the integer linear programming formulation, which includes a faulty virtual network reconfiguration ranking method based on weighted relative entropy, a hybrid multiple failures ranking algorithm, and a virtual node migration method based on weighted relative entropy. In the faulty virtual network reconfiguration ranking method based on weighted relative entropy and virtual node migration method based on weighted relative entropy, multiple ranking indicators are combined in a suitable way based on weighted relative entropy. In the hybrid multiple failures ranking algorithm, the virtual node and its connective virtual links are re-embedded, firstly. Evaluation results show that our heuristic method not only has the best acceptance ratio and normal operation ratio, but also achieves the highest long-term average revenue to cost ratio compared with other virtual network reconfiguration methods.

## 1. Introduction

Network virtualization (NV) allows multiple heterogeneous virtual networks (VNs) to be embedded onto the shared substrate network (SN), providing users with a variety of network services which has become one of the most promising technologies for future Internet [1,2,3]. It can decouple the network infrastructure and network services, and allow multiple heterogeneous VNs to share the SN resources through abstraction, distribution and isolation mechanism. In recent years, NV has been widely used in various fields, such as fog-supported software defined network (SDN) [4] and virtualized networked data centers [5]. One of the most challenging problems raised in NV is the virtual network embedding (VNE) problem, which has been known as the non-deterministic polynomial hard (NP-hard) problem [6].

In recent years, the SN failure events occur frequently. SN failures degrade the service performance and reliability of VNs [7]. SN failures include node failure and link failure. In network virtualization, multiple VNs are embedded onto the same SN and share the common substrate resources. Hence, substrate node failure causes virtual node and its adjacent virtual link failure. Substrate link failure causes virtual link failure. Also, there are single node/link failures and multiple node/link failures in SN which will cause complex and multiple virtual network (VN) failures [8]. Therefore, adopting suitable survivability strategies to against the increasingly complex network failures has become one of the main challenges in VN survivability.

A number of mechanisms have been proposed to improve the VN survivability [9]. These mechanisms can be broadly classified into two categories: backup mechanism [10] and reconfiguration mechanism [11]. The backup mechanism can also be classified into dedicated backup [12] and shared backup [13]. In shared backup mechanism, the backup resources can be shared with different VNs. In the dedicated case, the backup resources only can be used for dedicated VN. However, preallocating a backup resource is extremely expensive.

Instead, the reconfiguration mechanism can re-embed the faulty part of a VN without huge backup resource consumption. In reconfiguration mechanisms, backup resources are not preallocated, and the faulty parts of VNs are re-embedded after SN failures [14]. For instance, a topology awareness VN reconfiguration algorithm is proposed in [15]. The virtual node to be migrated is mapped onto the nearest mapped substrate node which is the neighbor mapping node of the migration virtual node. This algorithm takes topology indicator into consideration and improves the resource utilization. However, resource indicator is not considered in its node re-embedding strategy. To survive link failures, a novel recovery approach to restore VN without reserving backup resources prior to embedding is proposed in [16]. It not only considers the end-delay and delay variation requirements, but also takes actions to find a constrained shortest path between the nodes utilized by the failed VNs. The resource utilization is improved. However, this algorithm cannot deal with the problem of recovering VNs from node failures. As can be seen from above two algorithms, they cannot deal with the hybrid failures which include node failures and link failures.

To survive hybrid failures in SN, a heuristic survivable VNE based on node migration and link remapping is proposed in [17]. In VNE, the artificial bee colony algorithm is proposed to achieve an optimal solution. If the substrate node fails, the failed node is migrated to a normal node based on the greed rules, and the affected links are remapped based on the shortest path algorithm. Then, the shortest path is used to provide the best bandwidth principle to remap the affected links that connect with the failed node. This algorithm can deal with hybrid failures. However, the greed rules only take resource indicator into consideration and their re-embedding performance is limited. Also, they do not consider the hybrid failures in multiple faulty VNs.

To solve the problem of recovering a batch of VNs affected by a substrate node failure, a recovery approach for maximizing recovery and minimizing the cost of recovery is designed in [18]. An integer linear programming (ILP) formulation of this recovery scheme is provided, and a fast and scalable heuristic algorithm is also proposed to tackle the computational complexity of the ILP solution. It can recover a batch of VNs affected by a substrate node failure efficiently. However, it sorts the faulty VNs and virtual nodes based on the adjacent bandwidth resource, and it ignores the topology indicators. A generalized recovery approach that can achieve customized objectives is designed, and the corresponding ILP formulation is provided in [19]. Then, a fast and scalable heuristic algorithm to tackle the computational complexity of the ILP solution is proposed, and it is demonstrated that this heuristic algorithm has good recovery performance. However, it focuses on the problem of recovering from a node failure in VNE and ranks faulty VNs based on service level agreements. It cannot deal with the problem of recovering a batch of VNs affected by hybrid failures which include node and link failures. Also, in different recovery objects, recovering the faulty VNs based on service level agreements is not suitable.

As can be seen from the current research, the VN reconfiguration mechanisms focus on taking effective measures for network recovery after failures without preallocating resources. Although numerous efforts have been devoted, there are four obvious defects in existing VN reconfiguration algorithms. At first, VN reconfiguration for different types of failures in one VN and VN reconfiguration for single type of failures in multiple VNs are both studied. However, the VN reconfiguration for multiple types of failures in multiple VNs is not studied. Secondly, in reconfiguration of multiple faulty VNs, a single indicator is selected in most faulty VN ranking strategies. In different recovery objects, these strategies are not always suitable. Thirdly, a single resource indicator or topology indicator is taken into consideration in VN re-embedding, and the performance is limited. At last, the failures are not classified into certain types, and the recovery order of different types of failures is not researched.

In this paper, an ILP formulation of VN reconfiguration is provided, firstly. Then, the heuristic algorithm, called dynamic virtual network reconfiguration method for hybrid multiple failures based on weighted relative entropy (DVNRM-HMF-WRE), is proposed to solve the ILP formulation. In DVNRM-HMF-WRE, the faulty VN reconfiguration ranking method based on weighted relative entropy (FVNRRM-WRE), hybrid multiple failures ranking algorithm (HMFRA), and virtual node migration method based on weighted relative entropy (VNMM-WRE), are proposed. At last, five comparative experiments are set to demonstrate the performance of DVNRM-HMF-WRE.

The main contributions of this paper can be summarized as follows:

(1) We provide an ILP formulation of VN reconfiguration and propose the DVNRM-HMF-WRE to solve it. VNE is already intractable, and the combinatorial number of sequences of VNs in batch re-embedding further increases the complexity. Therefore, we only re-embed the faulty nodes and links without disrupting their unaffected parts in VN.
(2) We introduce a weighted relative entropy (WRE) method into FVNRRM-WRE and VNMM-WRE. In FVNRRM-WRE, we rank the faulty VNs with the help of multiple indicators, and combine them based on WRE. In VNMM-WRE, we use the WRE method to change the coefficients of node resource, and topology indicator to select the suitable candidate substrate node to reduce the resource consumption.
(3) We propose the HMFRA to handle the hybrid multiple failures. In HMFRA, faulty virtual node and its connective virtual links are handled first, which are the hardest the recover. HMFRA can make full use of the limited resources and improve the recovery performance.
(4) Five different simulation scenarios are conducted to validate the performance of DVNRM-HMF-WRE. The performance of DVNRM-HMF-WRE is compared with other algorithms in the first simulation experiment. Next, we simulate the impact of FVNRRM-WRE, HMFRA, and VNMM-WRE on the performance of DVNRM-HMF-WRE. At last, two factors are set to evaluate their influences on DVNRM-HMF-WRE.

The rest of this paper is organized as follows. Section 2 presents the models and evaluation indicators. In Section 3, we propose the ILP of VN reconfiguration. The DVNRM-HMF-WRE is given, and its details are shown in Section 4. In Section 5, we evaluate the proposed algorithm through extensive simulations and experiments. We conclude this paper with a summary and areas for future exploration in Section 6.

## 2. Models and Evaluation Indicators

In this section, we describe the models and evaluation indicators. Here, Table 1 summarizes the main notations used in this paper.

### 2.1. Network Model

#### 2.1.1. Substrate Network

The SN is modeled as a weighted undirected graph ***G***_S_ = (*N*_S_, *E*_S_), in which the substrate node set and substrate link set are represented by *N*_S_ and *E*_S_, respectively. In substrate nodes, the available CPU resource of substrate node *n*_s_ is denoted by *cpu*(*n*_s_), and the location attribute of substrate node *n*_s_ is denoted by *loc*(*n*_s_). Similarly, *bw*(*e*_s_) is taken to denote the available bandwidth resource of substrate link *e*_s_. Also, *N*_Sf_ represents faulty substrate node set and *E*_Sf_ represents the faulty substrate link set.

#### 2.1.2. Virtual Network

Similar to SN, the VN can be modeled as a weighted undirected graph ***G***_V_ = (*N*_V_, *E*_V_). *N*_V_ represents virtual node set and *E*_V_ represents the virtual link set. In virtual nodes, *cpu*(*n*_v_) denotes the required CPU resource of virtual node *n*_v_. In virtual links, *bw*(*e*_v_) is taken as the basic attribute to denote the required bandwidth resource of virtual link *e*_v_. *T*_V_ is used to denote the VN lifetime. ***G***_Vf_ represents the faulty VN set. Also, *N*_Vf_ represents faulty virtual node set and *E*_Vf_ represents the faulty virtual link set.

### 2.2. Failure Model

#### 2.2.1. Multiple Failures

The SN failures are denoted by a series of failure events *F_i,j_*, *I* = 1,2; *j* = 1, 2,...,*n*. *F*_1*,j*_ denotes the substrate node failure and *F*_1*,j*_∈*N*_Sf_. *F*_2*,j*_ denotes the substrate link failure and *F*_2*,j*_∈*E*_Sf_, *j* is the failure number. *t_s_*(*F_i,j_*) and *t_e_*(*F_i,j_*) denote the starting and ending time of *F_i,j_*, and *t_s_*(*F_i,j_*) < *t_e_*(*F_i,j_*). When ∃*j*1 < *j*2, if *t_s_*(*F_i,j_*_1_) < *t_s_*(*F_i,j_*_2_) < *t_e_*(*F_i,j_*_1_), multiple failures happen in SN. Multiple failures contain multiple node failures and multiple link failures.

#### 2.2.2. Failure Types

VN failures caused by SN failures can be divided into virtual node failures and virtual link failures. Also, virtual link failures can be divided into three categories. If virtual link takes faulty substrate node as the source or destination node, it is called VN link failure I. The virtual link which passes through the faulty substrate node but does not take it as the source or destination node, is called VN link failure II. The independent virtual link failure caused by independent substrate link failure is called VN link failure III.

#### 2.2.3. Hybrid Multiple Failures

If multiple failures occur at the same time and belong to multiple types, they are called hybrid multiple failures. 

**Figure 1:**
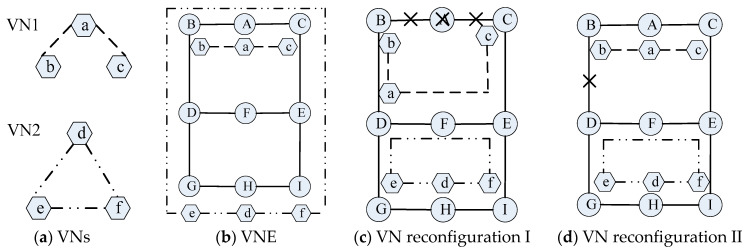
Examples of VN embedding and reconfiguration. (**a**) The VNs; (**b**) The process of VNE; (**c**) The process of VN reconfiguration for VN node failure, VN link failure I and II; (**d**) The process of VN reconfiguration for VN link failure III.

The process of VNE and VN reconfiguration are shown in Figure 1. Figure 1a shows two VNs, and Figure 1b illustrates an example for VNE problem. In VN1, the node embedding results are {a→A, b→B, c→C}, and link embedding results are {(a,b)→(A,B), (a,c)→(A,C)}, in which both the CPU and bandwidth requirements are satisfied. In VN2, the node embedding results are {d→H, e→G, f→I} and link embedding results are {(d,e)→(H,G), (d,f)→(H,I), (e,f) →(G,D,B,A,C,E,I)}.

Figure 1c,d illustrate the examples for VN reconfiguration. In Figure 1c, the substrate node A is faulty, which causes the connected substrate link (A,B) and (A,C) failure. The faulty virtual link (a,b) and (a,c) belongs to VN link failure I. Also, virtual link (e,f) in VN2 is faulty, which belongs to VN link failure II. At this time, virtual node a is re-embedded onto substrate node D. Virtual link (a,b) and (a,c) are re-embedded onto substrate link (D,B) and (D,F,E,C), respectively. After that, VN1 returns to normal. Virtual link (e,f) is re-embedded onto substrate link (G,D,F,E,I), then VN2 returns to normal.

In Figure 1d, substrate link (B,D) is faulty, which causes the virtual link (e,f) failure and this belongs to VN link failure III. After re-embedding virtual link (e,f) onto substrate link (G,D,F,E,I), VN2 returns to normal.

### 2.3. Evaluation Indicator

#### 2.3.1. Acceptance Ratio

The acceptance ratio (AR) is denoted by the number of VN requests which are embedded successfully, divided by the total number of VN requests. It is shown in Formula (1).

(1) $$r=\underset{T\to \infty }{\lim}\frac{\sum_{t=0}^{T}\left|VN_{\mathrm{map}}(t)\right|}{\sum_{t=0}^{T}\left|VN(t)\right|+\delta },$$

where *δ* is infinitely close to 0. |VN(t)| is the total number of VN requests at time *t* and $\left|VN_{\mathrm{map}}(t)\right|$ is the number of VNs which are embedded successfully at time *t*.

#### 2.3.2. Revenue to Cost Ratio

For VN request ***G***_V_ = (*N*_V_, *E*_V_), we denote revenue *R*(***G***_V_, *t*) and cost *C*(***G***_V_, *t*) as

(2) $$R(G_{V},t)=\alpha \sum_{n_{v}\in N_{V}}cpu(n_{v})+\sum_{e_{v}\in E_{V}}bw(e_{v}),$$

(3) $$C(G_{V},t)=\beta \sum_{n_{v}\in N_{V}}cpu(n_{v})+\sum_{e_{v}\in E_{V}}hops(e_{v})bw(e_{v}),$$

where *α* and *β* are weighting coefficients to balance CPU and bandwidth resources. In this paper, we set *α* = *β* = 1. Also, *hops*(*e*_v_) is the hop counts in substrate link corresponding to virtual link *e*_v_.

At time *t_i_*, if ***G***_V_ is faulty and can be re-embedded successfully, the revenue and cost will not change. Otherwise, the revenue and cost of ***G***_V_ are set to zero, and it also causes the penalty cost *PCost* which can be defined as

(4) $$PCost(G_{V},t)=\mu \cdot (t_{j}-t_{i})\cdot R(G_{V},t),$$

where *t_j_* is terminal time of ***G***_V_ and *μ* is the penalty coefficient. In this paper, *μ* = 2.

Long-term average revenue to cost ratio (LAR/CR) can be defined as

(5) $$\xi =\underset{T\to \infty }{\lim}\frac{\sum_{t=0}^{T}\sum_{G_{V}\subset VN_{\mathrm{map}}(t)}R\left(G_{V},t\right)-\sum_{t=0}^{T}\sum_{G_{V}\subset VN_{\mathrm{fr}}(t)}PCost\left(G_{V},t\right)}{\sum_{t=0}^{T}\sum_{G_{V}\subset VN_{\mathrm{map}}(t)}C\left(G_{V},t\right)},$$

where *VN*_fr_(*t*) is the set of faulty VNs which are re-embedded unsuccessfully. *VN*_map_(*t*) is the VNs which are embedded successfully at time *t*.

#### 2.3.3. Normal Operation Ratio

At time *t*, |*VN*_fr_(*t*)| denotes the number of faulty VNs which are unsuccessfully re-embedded. |*VN*(*t*)| is the total number of embedded VN requests. The normal operation ratio (NOR) can be defined as

(6) $$\eta (t)=\frac{\left|VN(t)\right|-\left|VN_{\mathrm{fr}}(t)\right|}{\left|VN(t)\right|}.$$

## 3. ILP of VN Reconfiguration

In this section, we formulate the ILP of VN reconfiguration. The objective function and constraints can be expressed as follows.

### 3.1. Objective Function

(7) $$\max\{\underset{T\to \infty }{\lim}\frac{\sum_{t=0}^{T}\sum_{G_{V}\subset VN_{\mathrm{map}}(t)}R\left(G_{V},t\right)-\sum_{t=0}^{T}\sum_{G_{V}\subset VN_{\mathrm{fr}}(t)}PCost\left(G_{V},t\right)}{\sum_{t=0}^{T}\sum_{G_{V}\subset VN_{\mathrm{map}}(t)}C\left(G_{V},t\right)}\}.$$

In this paper, our object is to get the maximum long-term average revenue to cost ratio. The corresponding variables in Formula (7) can be seen from Formulas (2)–(5) or Table 1.

### 3.2. Constraints

(8) $$\begin{array}{c} \forall n_{v}\in N_{V},\;\forall n_{s}\in N_{S},\\ x(n_{v},n_{s})=\left\{ \begin{array}{l} 1\;iff\;n_{v}\;\mathrm{is}\;\text{re-embedded}\;\mathrm{onto}\;n_{s}\\ 0\;\mathrm{otherwise} \end{array}\right.. \end{array}$$

(9) $$\begin{array}{c} \forall e_{up}\in E_{V},\;\forall e_{ij}\in E_{S},\\ x(e_{up},e_{ij})=\left\{ \begin{array}{l} 1\;iff\;e_{up}\;\mathrm{is}\;\text{re-embedded}\;\mathrm{onto}\;e_{ij}\\ 0\;\mathrm{otherwise} \end{array}\right.. \end{array}$$

(10) $$\begin{array}{c} \forall n_{v}\in N_{V},\;\forall n_{s}\in N_{S},\\ s(n_{v})=\left\{ \begin{array}{l} 1\;iff\;n_{v}\;\mathrm{is}\;\mathrm{faulty}\\ 0\;\mathrm{otherwise} \end{array}\right.,\;s(n_{s})=\left\{ \begin{array}{l} 1\;iff\;n_{s}\;\mathrm{is}\;\mathrm{faulty}\\ 0\;\mathrm{otherwise} \end{array}\right.. \end{array}$$

(11) $$\forall e_{v}\in E_{V},\;\forall e_{s}\in E_{S},\;s(e_{v})=\left\{ \begin{array}{l} 1\;iff\;e_{v}\;\mathrm{is}\;\mathrm{faulty}\\ 0\;\mathrm{otherwise} \end{array}\right.,\;s(e_{s})=\left\{ \begin{array}{l} 1\;iff\;e_{s}\;\mathrm{is}\;\mathrm{faulty}\\ 0\;\mathrm{otherwise} \end{array}\right..\;s(G_{V})=\left\{ \begin{array}{l} 1\;iff\;G_{V}\;\mathrm{is}\;\mathrm{faulty}\\ 0\;\mathrm{otherwise} \end{array}\right.,\;s(G_{S})=\left\{ \begin{array}{l} 1\;iff\;G_{S}\;\mathrm{is}\;\mathrm{faulty}\\ 0\;\mathrm{otherwise} \end{array}\right..$$

In constraint (8), if virtual node *n*_v_ is re-embedded onto substrate node n_s_, *x*(*n*_v_, *n*_s_) = 1. Otherwise, *x*(*n*_v_, *n*_s_) = 0. In constraint (9), if virtual link *e_up_* is re-embedded onto substrate link *e_ij_*, *x*(*e_up_*, *e_ij_*) = 1. Otherwise, *x*(*e_up_*, *e_ij_*) = 0. In constraint (10), if virtual node *n*_v_ is faulty, *s*(*n*_v_) = 1. Otherwise, *s*(*n*_v_) = 0. Also, if substrate node *n*_s_ is faulty, *s*(*n*_s_) = 1. Otherwise, *s*(*n*_s_) = 0. In constraint (11), if virtual link *e*_v_ is faulty, *s*(*e*_v_) = 1. Otherwise, *s*(*e*_v_) = 0. If substrate link *e*_s_ is faulty, *s*(*e*_s_) = 1. Otherwise, *s*(*e*_s_) = 0. If virtual network ***G***_V_ is faulty, *s*(***G***_V_) = 1. Otherwise, *s*(***G***_V_) = 0. If substrate network ***G***_S_ is faulty, *s*(***G***_S_) = 1. Otherwise, *s*(***G***_S_) = 0.

(12) $$\forall n_{v}\in N_{V},\;\forall n_{s}\in N_{S},\;cpu(n_{v})\times x(n_{v},n_{s})\leq cpu(n_{s}),$$

(13) $$\forall e_{up}\in E_{V},\;\forall e_{ij}\in E_{S},\;\sum_{\forall e_{up}\in E_{V}}bw(e_{up})\times x(e_{up},e_{ij})\leq bw(e_{ij}).$$

Constrains (12) and (13) are resource constraints, and they ensure that if virtual node *n*_v_ and virtual link *e_up_* are selected to re-embed, the candidate substrate node *n*_s_ and substrate link *e_ij_* should have more resources than the virtual node *n*_v_ and virtual link *e_up_* request, respectively.

(14) $$x(n_{v},n_{s})\times dis(loc(n_{s}),loc(n_{v}))\leq D(n_{s}).$$

Constrain (14) is location constraint and *dis*(*loc*(*n*_s_), *loc*(*n*_v_)) denotes the distance between substrate node *n*_s_ and virtual node *n*_v_.

(15) $$\forall n_{v}\in N_{V},\;\forall n_{s}\in N_{S},\;\forall e_{up}\in E_{V},\;\forall e_{ij}\in E_{S},\;\begin{array}{l} \sum_{e_{ji}\in E_{S}}x(e_{up},e_{ji})-\sum_{e_{ij}\in E_{S}}x(e_{up},e_{ij})\\ =\left\{ \begin{array}{ll} 1, & x(n_{u},n_{j})=1\\ -1, & x(n_{u},n_{j})=1\\ 0, & \mathrm{otherwise} \end{array}\right. \end{array}$$

Constrain (15) is the connectivity constraint. It refers to the flow conservation constraint for routing one unit of net flow from virtual node *n_u_* to virtual node *n*_p_.

(16) $$\forall n_{s}\in N_{S},\;\sum_{n_{v}\in N_{V}}x(n_{v},n_{s})\leq 1\;$$

(17) $$\forall n_{v}\in N_{V},\;\sum_{n_{s}\in N_{S}}x(n_{v},n_{s})=1\;$$

Constrains (16) and (17) are re-embedding constraints. Constrain (16) ensures that all virtual nodes in same VN are re-embedded on different substrate nodes. Constrain (17) ensures that each virtual node is re-embedded on up to one substrate node.

(18) $$\mathrm{if}\;\forall n_{v}\in N_{V}\mathrm{and}\;s(n_{v})=1,\;\mathrm{then}\;s(G_{V})=1\;$$

(19) $$\mathrm{if}\;\forall e_{up}\in E_{V}\mathrm{and}\;s(e_{up})=1,\;\mathrm{then}\;s(G_{V})=1\;$$

(20) $$\forall n_{i}\in N_{S},\;\forall n_{j}\in N_{S},\;\forall e_{ij}\in E_{S},\;\mathrm{if}\;s(n_{i})=1,\;\mathrm{then}\;s(e_{ij})=1\;$$

(21) $$\forall n_{q}\in N_{\mathrm{Vf}},\;\forall n_{j}\in N_{\mathrm{Sf}},\;x(n_{q},n_{j})=0\;$$

(22) $$\forall e_{up}\in E_{\mathrm{Vf}},\;\forall e_{ij}\in E_{\mathrm{Sf}},\;x(e_{up},e_{ij})=0\;$$

Constrains (18)–(22) are failure constraints. Constrains (18) and (19) ensure that if either the virtual node or virtual link fail in VN, the VN will also fail. Constrain (20) ensures that if one of the end-nodes in substrate link fails, the substrate link will fail. Constrain (21) ensures that virtual node and the corresponding substrate node re-embedded by it cannot fail at the same time. Constrain (22) ensures that virtual link and the corresponding substrate link re-embedded by it cannot fail at the same time.

This ILP is known to be NP-hard problem, and solving it is computationally intractable. Even though optimal results can be obtained by some exact algorithms or open source linear programming toolkit GLPK, they may incur exponential increasing running time. Consequently, they cannot be scaled to address large-scale VNs. Hence, most researchers solve the ILP problem of VN reconfiguration by proposing a corresponding heuristic algorithm which has short computational time and gets an approximate optimal solution. Therefore, we propose a novel heuristic algorithm called DVNRM-HMF-WRE to solve the ILP of VN reconfiguration.

## 4. DVNRM-HMF-WRE

### 4.1. Problem Statement

The VN reconfiguration is a complex and dynamic process. There are many event statements at the same time. We analyze the possible event statements and provide the dynamic process of VN reconfiguration first. Then, three algorithms in DVNRM-HMF-WRE are proposed. 

VN embeds and leaves dynamically. Many types of failures occur randomly in SN. At time t, there are four possible event statements:

1. When the lifetime of VN ends, the corresponding SN resources are released.
2. When the faulty component in SN is repaired, its resources also return to normal.
3. When SN failure occurs, the resources of faulty component are set to zero and the VN embedded on it needs to be re-embedded. If multiple failures occur at the same time, the faulty VNs will be re-embedded one by one. When all faulty virtual components in one VN are re-embedded successfully, the VN reconfiguration is successful.
4. When VN request arriving, VN begins to embed. If succeed, the corresponding resources are used.

**Figure 2:**
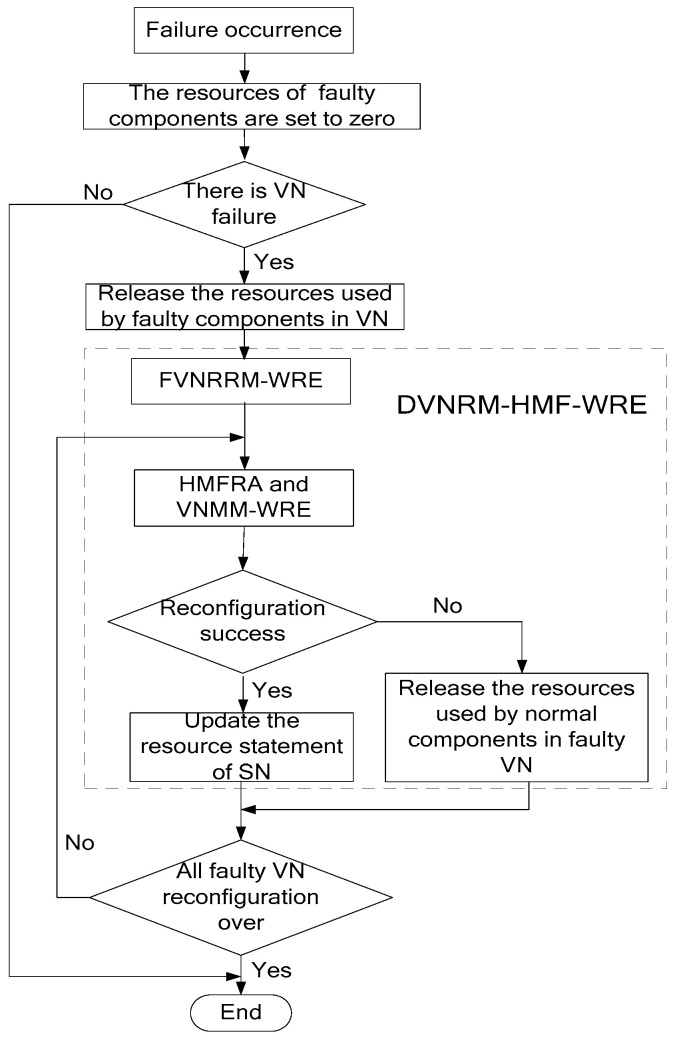
The process of DVNRM-HMF-WRE.

When multiple SN failures occur, the process of DVNRM-HMF-WRE is shown in Figure 2.

As can be seen that, from Figure 2, when failure occurs, the resources of faulty components are set to zero. If there are some VNs embedded onto the faulty components, the resources used by faulty components in VNs are released. Then, the DVNRM-HMF-WRE is used to re-embed the faulty VNs. In DVNRM-HMF-WRE, the FVNRRM-WRE is used to rank multiple faulty VNs to decide which faulty VN should be re-embedded first. The HMFRA and VNMM-WRE are used together to recover the hybrid multiple failures in one VN. The HMFRA is proposed to rank hybrid multiple faulty nodes/links in one VN, and the VNMM-WRE is provided to improve the efficiency of virtual node migration. If the VN reconfiguration is successful, the resource statement of SN I updated, otherwise, the resources used by normal components in faulty VN are released. If all VN reconfigurations are over, the DVNRM-HMF-WRE is stopped. 

### 4.2. FVNRRM-WRE

When multiple failures occur at the same time, and substrate resources are limited, the reconfiguration sequence of faulty VNs is especially important. In faulty VN ranking strategies, ranking indicators and ranking method are very important. Among them, faulty component number, VN revenue, and remaining lifetime of the faulty VN all have obvious impact on the reconfiguration performance. They are selected to rank the faulty VNs. When ranking faulty VNs, a fixed formula is usually used, and it cannot adapt to the changing environment. Therefore, the WRE method is introduced in this paper.

The WRE method is a classical ranking method which can adjust the indicator coefficients to adapt the changing environment. It is improved by the classical technique for order preference by similarity to an ideal solution (TOPSIS) [20]. In TOPSIS, the generalized distance is unable to distinguish the point in perpendicular bisector between positive and negative ideal solution [21]. Therefore, the relative entropy is used, instead, to the generalized distance.

For system A and B in state *A_i_* and *B_i_* (*i* = 1, 2,…,*N*), their relative entropy, *C*, can be defined by

(23) $$C=\sum_{i=1}^{N}\left[A_{i}\log\frac{A_{i}}{B_{i}}+(1-A_{i})\log\frac{1-A_{i}}{1-B_{i}}\right].$$

There are N faulty VNs, and each faulty VN has M evaluation indicators. The *j*-th indicator coefficient of the *i*-th node is denoted as *X_ij_* (*i* = 1, 2, …, *N*; *j* = 1, 2, …, *M*), and all coefficients of faulty VNs constitute a decision matrix ***X*** which is denoted as

(24) $$X=\left(\begin{array}{cccc} x_{11} & x_{12} & \cdots & x_{1M}\\ x_{21} & x_{22} & \cdots & x_{2M}\\ \vdots & \vdots & \ddots & \vdots \\ x_{N1} & x_{N2} & \cdots & x_{NM} \end{array}\right).$$

Also, the coefficient is normalized to make a fair comparison between different indicators.

In different environments, each indicator has a different importance. The weighting coefficient of the *j*-th indicator is expressed as $\omega_{j}$(j=1,2,…,M, $\sum \omega_{j}=1$), and the weighted normalized decision matrix is denoted by

(25) $$Y=X\omega =\left(\begin{array}{cccc} x_{11}\omega_{1} & x_{12}\omega_{2} & \cdots & x_{1M}\omega_{M}\\ x_{21}\omega_{1} & x_{22}\omega_{2} & \cdots & x_{2M}\omega_{M}\\ \vdots & \vdots & \ddots & \vdots \\ x_{N1}\omega_{1} & x_{N2}\omega_{2} & \cdots & x_{NM}\omega_{M} \end{array}\right).$$

The positive and negative ideal solutions *A*^+^ and *A*^−^ are determined as

(26) $$A^{+}=\{\max(y_{i1},y_{i2},\ldots ,y_{iM})\}=\{y_{1}^{\max},y_{2}^{\max},\ldots ,y_{M}^{\max}\},$$

(27) $$A^{-}=\{\min(y_{i1},y_{i2},\ldots ,y_{iM})\}=\{y_{1}^{\min},y_{2}^{\min},\ldots ,y_{M}^{\min}\}.$$

The relative entropies of each solution to positive and negative ideal solution are calculated as

(28) $$C_{i}^{+}=\sum_{j=1}^{M}\left[y_{j}^{\max}\log\frac{y_{j}^{\max}}{y_{ij}}+(1-y_{j}^{\max})\log\frac{1-y_{j}^{\max}}{1-y_{ij}}\right],$$

(29) $$C_{i}^{-}=\sum_{j=1}^{M}\left[y_{j}^{\min}\log\frac{y_{j}^{\min}}{y_{ij}}+(1-y_{j}^{\min})\log\frac{1-y_{j}^{\min}}{1-y_{ij}}\right].$$

The similarity between each solution and the ideal one is calculated as

(30) $$Z_{i}=\frac{C_{i}^{-}}{C_{i}^{-}+C_{i}^{+}},0\leq Z_{i}\leq 1.$$

The details of FVNRRM-WRE method are described in Algorithm 1.

**Algorithm 1.** The FVNRRM-WRE**Input**: ***X***, weighting coefficients**Output**: *Z_i_*1. Bring ***X*** and the weighting coefficient of each indicator into Formula (25) to construct the weighted normalized matrix ***Y***.2. Calculate the positive and negative ideal scheme *A*^+^ and *A*^−^.3. Calculate the positive and negative ideal solution *C*^+^ and *C*^−^.4. Calculate *Z_i_* and get the importance degrees of faulty VNs.

In Algorithm 1, ***X*** is the multiple indicators decision matrix which includes three indicators, called faulty components number, VN revenue, and remaining lifetime of the faulty VN. To calculate Z_i_, which is the importance degree of faulty VN_i_, the WRE method is widely used based on Formulas (26)–(30). After getting the importance degrees of faulty VNs, rank the faulty VNs in order of importance degree, from large to small, which can re-embed the important VN first.

### 4.3. HMFRA

In a faulty VN, there are multiple failures which include node failures and link failures. Recovery sequence is important in VN reconfiguration, and the HMFRA is proposed to recover different types of failures in a suitable manner. The details of HMFRA are shown in Algorithm 2.

**Algorithm 2.** The HMFRA**Input**: ***G***_S_, ***G***_V_, the set of faulty node, the set of VN link failure I, the set of VN link failure II, the set of VN link failure III**Output**: ***G***_S_, ***G***_V_1. if  the faulty node set is not empty2.   Migrate the virtual nodes using VNMM-WRE and re-embed all their connective links (VN   link failures I) using *k*-shortest path algorithm.3.   if  all virtual nodes are migrated and all their adjacent links are re-embedded successfully4.     Update ***G***_S_ and ***G***_V_5.   if  the set of VN link failure II is not empty 6.     Re-embed all VN link failures II7.    if  all VN link failures II are re-embedded successfully8.      Update ***G***_S_ and ***G***_V_9.      if  the set of VN link failure III is not empty 10.       Re-embed all of them 11.      if  all VN link failures III are re-embedded successfully12.         Update ***G***_S_ and ***G***_V_13.         Return VN_RE-EMBED_SUCCESS14.       else  release the resources in ***G***_S_ which are used by the normal parts 15.         Update ***G***_S_16.         Return VN_RE-EMBED_FAILURE17.       end if18.      else  update ***G***_S_ and ***G***_V_19.        Return VN_RE-EMBED_SUCCESS 20.      end if21.    else  release the resources in ***G***_S_ which are used by the normal parts 22.      Update ***G***_S_23.      Return VN_RE-EMBED_ FAILURE24.    end if25.   else  update ***G***_S_ and ***G***_V_26.       Return VN_RE-EMBED_SUCCESS 27.   end if28.  else  release the resources in ***G***_S_ which are used by the normal parts 29.      Update ***G***_S_30.      Return VN_RE-EMBED_ FAILURE31.  end if32. else 33.   if  the set of VN link failure III is not empty 34.     Re-embed all of them 35.    if  all VN link failures III are re-embedded successfully36.       Update ***G***_S_ and ***G***_V_37.       Return VN_RE-EMBED_SUCCESS38.    else  release the resources in ***G***_S_ which are caused by the normal parts 39.      Update ***G***_S_40.      Return VN_RE-EMBED_ FAILURE41.    end if42.  end if43. end if

As can be seen from Algorithm 2:

1. Faulty virtual node and its connective links (VN link failures I) are recovered first. If substrate node is faulty, virtual node and its connective virtual links both need to be re-embedded. They will consume huge substrate node and link resources. They are the hardest to re-embed. If this succeeds, go on re-embedding virtual links in the set of VN link failure II. Otherwise, VN reconfiguration has failed.
2. If the VN link failures II are re-embedded successfully, then judge if there are VN link failures III. If there are VN link failures III, re-embed them. If this succeeds, VN reconfiguration is successful.
3. If there is no VN link failure III after re-embedding the VN link failure II successfully, the faulty VN returns to normal. If the VN link failure II fails to re-embed, the faulty VN reconfiguration has failed.
4. If there is only VN link failure III, re-embed these faulty virtual links. If this succeeds, VN reconfiguration is successful.

### 4.4. VNMM-WRE

In HMFRA, the method of faulty virtual node migration is called VNMM-WRE, which is proposed in this section and the method of virtual link re-embedding is *k*-shortest path algorithm [22].

Similar to FVNRRM-WRE, the WRE method is also introduced into VNMM-WRE. In virtual node ranking, the importance degree of each virtual node *Z*(*n*_v_) is calculated using WRE method, which selects node CPU resource and node adjacent link bandwidth resource as the indicators. In substrate node ranking, the importance degree of each substrate node *Z*(*n*_s_) is calculated using WRE method which selects node CPU resource, node adjacent link bandwidth resource and the reciprocal of hop counts between candidate substrate node and substrate node which is embedded by the neighboring virtual node of the migrated virtual node as the indicators [23].

The details of VNMM-WRE are shown in Algorithm 3

**Algorithm 3.** The VNMM-WRE**Input:** Faulty virtual nodes which need to migrate**Output:** NodeMigraingList1. for  each virtual node *n*_v_∈*N*_V_2.   Calculate the *Z*(*n*_v_)3. end for4. Rank virtual nodes in order of *Z*(*n*_v_) from large to small 5. Save virtual node ranking order into *VirtualNodeList*6. for each virtual node in *VirtualNodeList*7.   Select the candidate substrate node set *Can*(*n*_v*i*_) which satisfies the resource constraints8.   Remove the embedded substrate nodes from *Can*(*n*_v*i*_)9.     if  *Can*(*n*_v*i*_) is empty 10.     Return NODE_ MIGRATE_FAILURE11.   else12.     for  each candidate node *n*_s_ in *Can*(*n*_v*i*_) 13.      Calculate the *Z*(*n*_s_) of each substrate node in *Can*(*n*_v*i*_)14.     end for15.     for each virtual node in *Can*(*n*_v*i*_)16.      Re-embed *n*_v_ to *n*_s_ which has the largest *Z*(*n*_s_) value17.      Re-embed the virtual links which connect the virtual node18.       if all virtual links are re-embedded successfully19.         Save the substrate node *n*_s_ into *NodeMigratingList*20.         break21.       else22.         Remove the virtual node *n*_v_ from *Can*(*n*_v*i*_)23.      end if24.      end for25.      Update ***G***_S_ and ***G***_V_26.     Return NODE_MIGRATE_SUCCESS27.   end if28. end for

In Algorithm 3:

1. Lines 1–5: calculate the *Z*(*n*_v_) of each faulty virtual node and rank them based on *Z*(*n*_v_). Then save the ranking results into *VirtualNodeList*.
2. Lines 6–14: select the candidate substrate nodes and calculate their *Z*(*n*_s_).
3. Lines 15–28: re-embed the virtual node and its adjacent virtual links.

In summary, with the help of FVNRRM-WRE, HMFRA, and VNMM-WRE, the faulty VNs caused by hybrid multiple failures can be recovered.

### 4.5. COMPLEXITY ANALYSIS

The DVNRM-HMF-WRE includes FVNRRM-WRE, HMFRA, and VNMM-WRE. In FVNRRM-WRE, all faulty VNs are ranked based on the WRE method. Its complexity is $O(\left|N_{\mathrm{FN}}\right|)$, in which $\left|N_{\mathrm{FN}}\right|$ is the total number of faulty VNs. The HMFRA and VNMM-WRE are used, together, to recover the hybrid multiple failures in VNs. In the recovery of faulty VNs, the complexity of virtual node re-embedding is $O(\left|N_{V}\right|{\left|N_{S}\right|}^{2})$. $\left|N_{V}\right|$ is the total number of faulty virtual nodes in all faulty VNs and $\left|N_{S}\right|$ is the total number of substrate nodes. The virtual link re-embedding algorithm is *k-*shortest path algorithm and its complexity is $O(k(\left|N_{V}\right|+\left|E_{V}\right|)(\left|E_{S}\right|+\left|N_{S}\right|\mathrm{lg}\left|N_{S}\right|))$. $\left|E_{V}\right|$ represents the total number of faulty virtual links in all faulty VNs and $\left|E_{S}\right|$ represents the total number of substrate links. Therefore, the total complexity of DVNRM-HMF-WRE is $O(\left|N_{\mathrm{FN}}\right|+\left|N_{V}\right|{\left|N_{S}\right|}^{2}+k(\left|N_{V}\right|+\left|E_{V}\right|)(\left|E_{S}\right|+\left|N_{S}\right|\mathrm{lg}\left|N_{S}\right|))$.

## 5. Simulation

In this section, we present the performance evaluation of the proposed DVNRM-HMF-WRE. 

### 5.1. Simulation Setup

We run our experiments on a workstation with Lenovo Tianyi 510Pro (Lenovo, Beijing, China) with Windows 10 system (Microsoft Corporation, Redmond, WA, USA). The hardware platform is the Inter Core i7-7700 3.6 GHz process (Intel Corporation, Santa Clara, CA, USA) with 8 GB RAM (Intel Corporation, Santa Clara, CA, USA). The software is MATLAB R2007a (MathWorks, Natick, MA, USA). We run our simulations for 3000 time units to leave the performance in a stable state. Each simulation is performed 10 times, and we take the average values as the final results. Simulation codes and results could be found in the Supplementary File.

The SN topology and VN topology used in simulation are generated by the improved Salam network topology random generation algorithm [24]. It is worth noting that, the main parameters of VN, SN, and failures are summarized in Table 2. In Table 2, [*xmin, xmax*] denotes a uniform distribution between *xmin* and *xmax*. *Pois*{*p*} and *Expo*{*g*} stand for the Poisson and exponential distributions, with mean *p* and *g*, respectively.

### 5.2. Scenarios

Five scenarios are set in our simulation to validate the performance of DVNRM-HMF-WRE. 

In the first scenario, the performance of DVNRM-HMF-WRE is compared with two typical VN reconfiguration methods TA-VNR [15] and ReNoVatE [18]. 

In the second scenario, our FVNRRM-WRE is compared with the other three faulty VN ranking methods to evaluate the impact of the FVNRRM-WRE. The details of them are shown in Table 3. 

In the third scenario, our HMFRA is compared with the other two failure handling methods to evaluate the impact of the HMFRA. The details of them are shown in Table 4. 

In the fourth scenario, our VNMM-WRE is compared with the other two VN node migration methods which include DVNRM-HMF-HOPS and DVNRM-HMF-RES. The details of them are shown in Table 5. 

At last, the arrival rate of failure, *u*, and the lifetime of failure, *T*, are set to evaluate their effects on DVNRM-HMF-WRE.

### 5.3. Simulation Results

#### 5.3.1. Comparison of Different VN Reconfiguration Methods

**Figure 3:**
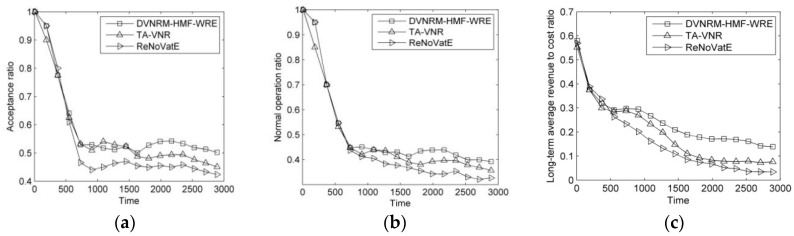
Comparison of DVNRM-HMF-WRE, TA-VNR and ReNoVatE. (**a**) Acceptance ratio; (**b**) Normal operation ratio; (**c**) Long-term average revenue to cost ratio.

The results of comparison of faulty VN reconfiguration methods are shown in Figure 3.

As can be seen from Figure 3a–c, the DVNRM-HMF-WRE proposed in this paper has the best AR, NOR, and LAR/CR. It achieves 5.7% and 11.3% higher AR than TA-VNR and ReNoVatE, respectively. Its NOR is 6.9% and 11% higher than TA-VNR and ReNoVatE, respectively. Its LAR/CR is 25.7% and 41.9% higher than TA-VNR and ReNoVatE, respectively. In DVNRM-HMF-WRE, faulty components number, VN revenue, and remaining lifetime of faulty VN are all considered to rank the faulty VNs. The resource and topology indicators are both used based on WRE in VNMM-WRE. In addition, the faulty virtual node and VN link failures I are recovered first. Therefore, the performance of DVNRM-HMF-WRE is the best.

#### 5.3.2. Comparison of Faulty VN Ranking Methods

**Figure 4:**
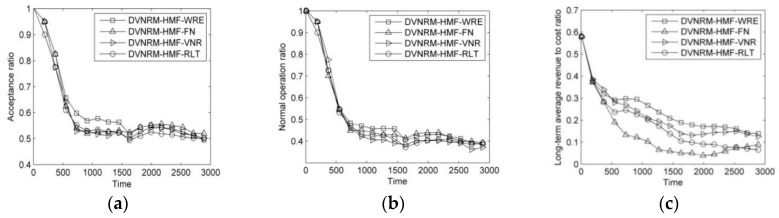
Comparison of different faulty VN ranking methods. (**a**) Acceptance ratio; (**b**) Normal operation ratio; (**c**) Long-term average revenue to cost ratio.

The results of comparison of faulty VN ranking methods are shown in Figure 4.

As can be seen from Figure 4a–c, the DVNRM-HMF-WRE achieves 4.5%, 5.3%, and 5.6% higher AR than DVNRM-HMF-FN, DVNRM-HMF-VNR, and DVNRM-HMF-RLT, respectively. The DVNRM-HMF-WRE achieves 3.1%, 6.4%, and 5.1% higher NOR than DVNRM-HMF-FN, DVNRM-HMF-VNR, and DVNRM-HMF-RLT, respectively. The DVNRM-HMF-WRE achieves 31.1%, 17.4%, and 25.1% higher LAR/CR than DVNRM-HMF-FN, DVNRM-HMF-VNR, and DVNRM-HMF-RLT, respectively. In this paper, our objective is to maximize the LAR/CR. If re-embedding the faulty VN fails, a high penalty cost will be paid. Improving the proportion of VN revenue, remaining lifetime of the faulty VN, and re-embedding the faulty VN which has higher penalty cost and revenue based on WRE, can improve the LAR/CR significantly.

#### 5.3.3. Comparison of Failure Handling Methods

**Figure 5:**
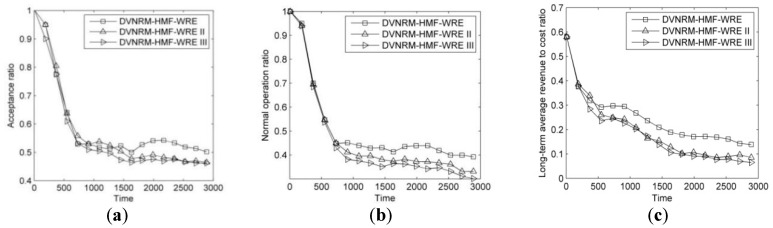
Comparison of different failure handling methods. (**a**) Acceptance ratio; (**b**) Normal operation ratio; (**c**) Long-term average revenue to cost ratio.

The results of comparison of failure handling methods are shown in Figure 5.

As can be seen from Figure 5a–c, the performance of DVNRM-HMF-WRE is better than the other two failure handling methods. It achieves 5.7% and 7.3% higher AR than DVNRM-HMF-WRE II and DVNRM-HMF-WRE III, respectively. Its NOR is 8.5% and 12.8% higher than DVNRM-HMF-WRE II and DVNRM-HMF-WRE III, respectively. Its LAR/CR is 24% and 27.9% higher than DVNRM-HMF-WRE II and DVNRM-HMF-WRE III, respectively. When the substrate node is faulty, the virtual node and its connective virtual links (VN link failure I) will fail at the same time. Substrate node and link resources are both consumed to re-embed the faulty VN. Compared with the other two link failures, node failure is the hardest to recover. Therefore, re-embedding faulty virtual node and its connective virtual links, first, can improve the performance of VN recovery algorithm. 

#### 5.3.4. Comparison of Different VN Node Migration Methods

**Figure 6:**
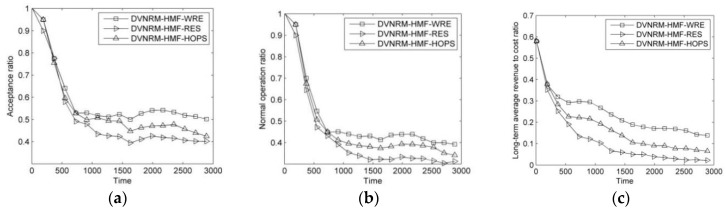
Comparison of different VN node migration methods. (**a**) Acceptance ratio; (**b**) Normal operation ratio; (**c**) Long-term average revenue to cost ratio.

The results of comparison of VN node migration methods are shown in Figure 6.

As can be seen from Figure 6a,b, DVNRM-HMF-WRE has the best AR and NOR. It achieves approximately 8.3% and 16.3% higher AR than VNMM-HMF-HOPS and VNMM-HMF-RES, respectively. The NOR of VNMM-HMF-WRE is approximately 7.4% and 17.4% higher than VNMM-HMF-HOPS and VNMM-HMF-RES, respectively. In Figure 6c, the advantage of VNMM-WRE is more obvious in LAR/CR. It is approximately 31.8% and 68.3% higher than VNMM-HMF-HOPS and VNMM-HMF-RES, respectively. In VNMM-HMF-WRE, the resource and topology indicators are both used to reduce substrate resource consumption in VN reconfiguration. Also, WRE is introduced to calculate the candidate substrate node importance. 

#### 5.3.5. The Effect of Arrival Rate of Failure and Lifetime of Failure on DVNRM-HMF-WRE

In this section, the arrival rate of failure *u* and the lifetime of failure *T* are selected to describe their effects on DVNRM-HMF-WRE. 

(1) The Arrival Rate of Failure *u*

**Figure 7:**
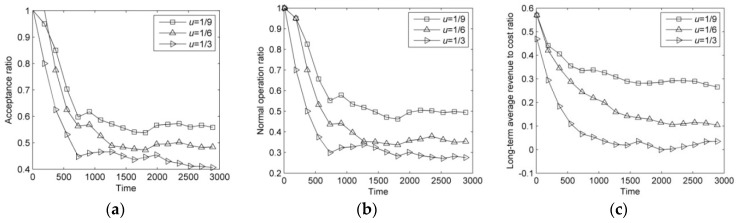
Comparison of DVNRM-HMF-WRE in different arrival rates of failure. (**a**) Acceptance ratio; (**b**) Normal operation ratio; (**c**) Long-term average revenue to cost ratio.

In this section, the arrival rates of failure are set to 1/3, 1/6, and 1/9. The results of DVNRM-HMF-WRE in different arrival rates of failure are shown in Figure 7.

As can be seen from Figure 7a–c, with the increase of arrival rate of failure *u*, the number of SN failures keeps increasing, which results in a reduction in resources used for VN embedding and reconfiguration. Therefore, the AR and NOR of VN are decreasing. A large number of penalty cost caused by the failure of VN reconfiguration significantly reduces the LAR/CR. 

(2) The Lifetime of Failure *T*

**Figure 8:**
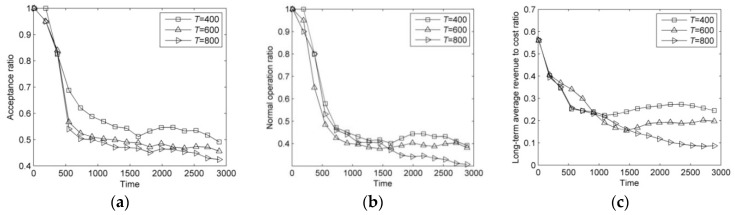
Comparison of DVNRM-HMF-WRE performance in different arrival rates of failure. (**a**) Acceptance ratio; (**b**) Normal operation ratio; (**c**) Long-term average revenue to cost ratio.

In this section, the lifetimes of failure are set to 400, 600, and 800. The results of DVNRM-HMF-WRE in different lifetimes of failure are shown in Figure 8.

As can be seen from Figure 8a–c, increasing the lifetime of failure can reduce the available substrate resources, which not only decreases the AR, but also results in a reduction in resources used for VN reconfiguration. Therefore, with the increase of the lifetime of failure, the AR, NOR, and LAR/CR are all reduced significantly.

## 6. Conclusions and Future Work

In this paper, we have addressed the problem of recovering faulty VNs affected by hybrid multiple failures in SN. In this regard, we have formulated the VN reconfiguration to an ILP model to maximize the LAR/CR. We have proposed an efficient heuristic policy called DVNRM-HMF-WRE to solve this ILP model. In DVNRM-HMF-WRE, the WRE method is introduced into FVNRRM-WRE and VNMM-WRE. In FVNRRM-WRE, the WRE method is used to rank the multiple faulty VNs. In VNMM-WRE, candidate substrate node are selected based on WRE method which takes resource and topology indicators into consideration. Also, a failure handling method called HMFRA is proposed, which handles virtual node failure and VN link failure I first. At last, five experiments are designed. The first experiment verifies that the proposed DVNRM-HMF-WRE has excellent performance than other typical VN reconfiguration methods. The next three experiments assess that our proposed faulty VN ranking method, failure handling method, and VN node migration method perform better than other corresponding methods. The last experiment sets two different scenarios to evaluate the performance of DVNRM-HMF-WRE in different arrival rates and lifetimes of failures. Evaluation results show that decreasing the arrival rate and the lifetime of failure can improve the performance of VN reconfiguration.

In future research, we will introduce the privacy and energy consumption into VN reconfiguration. Without adequate protection, users from a VN might be able to gain unauthorized access to data being transmitted through other VNs, violating the privacy of the entities that own those networks. Hence, privacy-oriented VNE and preserving privacy with VN stacks gradually get more and more attention. Also, energy consumption has become another hot topic in NV. With the increase of communication traffic every year, some energy models have been proposed to minimize the energy consumption in NV, such as the load-dependent power consumption model in VNE, the energy-efficient resource management for real-time service function chain in fog-supported SDN and network-aware energy optimization model for VN. Therefore, the ILP formulation and the heuristic algorithm could be extended to consider the privacy and energy consumption in future work.

## Figures and Tables

**Table 1:** Notations.

Notation	Description	Notation	Description
***G***_S_	SN	***G***_V_	VN
*N*_S_	Substrate node set	*N*_V_	Virtual node set
*E*_S_	Substrate link set	*E*_V_	Virtual link set
*|N*_S_|	Number of substrate nodes	*|N*_V_|	Number of virtual nodes
*|E*_S_|	Number of substrate links	*|E*_V_|	Number of virtual links
*n*_s_	Substrate node	*n*_v_	Virtual node
*e*_s_	Substrate link	*e*_v_	Virtual link
*cpu*(*n*_s_)	CPU resource of *n*_s_	*cpu*(*n*_v_)	Required CPU resource of *n*_v_
*bw*(*e*_s_)	Bandwidth resource of *e*_s_	*bw*(*e*_v_)	Required bandwidth resource of *e*_v_
*loc*(*n_s_*)	Location attribute of *n*_s_	*N*_Vf_	Faulty virtual node set
*N_S_*_f_	Faulty substrate node set	*E*_Vf_	Faulty virtual link set
*E_S_*_f_	Faulty substrate link set	*T*_V_	Lifetime of VN
***G***_Vf_	Faulty VN set	*F_i,j_*	Failure events
*F*_1*,j*_	Substrate node failure	*F*_2*,j*_	Substrate link failure
*t_e_*(*F_i,j_*)	Ending time of *F_i,j_*	*t_s_*(*F_i,j_*)	Starting time of *F_i,j_*
*VN*_map_(*t*)	VNs which are embedded successfully at time *t*	*|VN*_map_(*t*)|	Number of VNs which are embedded successfully at time *t*
*R*(***G***_V_, *t*)	Revenue of ***G***_V_ at time *t*	*VN*_fr_(*t*)	Set of faulty VNs which are re-embedded unsuccessfully
*C*(***G***_V_*, t*)	Cost of ***G***_V_ at time *t*	*PCost*(***G***_V_, *t*)	Penalty cost of ***G***_V_ at time *t*
*hops*(*e*_v_)	Hop counts in substrate link corresponding to virtual link *e*_v_	*|VN_fr_*(*t*)|	Number of faulty VNs which are re-embedded unsuccessfully
*|VN*(*t*)|	Total number of VN requests at time *t*	*η*(*t*)	Normal operation ratio at time *t*
*x*(*n_v_,n_s_*)∈{0,1}	*x*(*n*_v_, *n*_s_) = 1 if virtual node *n*_v_ is embedded onto substrate node *n*_s_	*x*(*e_up_*, *e_ij_*)∈{0,1}	*x*(*e_up_*, *e_ij_*) = 1 if virtual link *e_up_* is embedded onto substrate link e*_ij_*
*s*(*n*_v_)∈{0,1}	s(*n*_v_) = 1 if *n*_v_ is faulty	*s*(*n*_s_)∈{0,1}	s(*n*_s_) = 1 if *n*_s_ is faulty
*s*(*e*_v_)∈{0,1}	s(*e*_v_) = 1 if *e*_v_ is faulty	*s*(*e*_s_)∈{0,1}	s(*e*_s_) = 1 if *e*_s_ is faulty
*s*(***G***_V_) ∈{0,1}	s(***G***_V_) = 1 if ***G***_V_ is faulty	*s(**G***_S_)∈{0,1}	s(***G***_S_) = 1 if ***G***_S_ is faulty
*dis*(*loc*(*n*_s_)*, loc*(*n*_v_))	Distance between *n_s_* and *n_v_*	*A_i_*	State of system A
*B_i_*	State of system B	*C*	Entropy value
*X_ij_*	The *j-*th indicator coefficient of the *i*th node	***X***	Decision matrix
*ω*_j_	Weighting coefficient of the *j-*th indicator	*A^+^*	Positive ideal solutions
*A^−^*	Negative ideal solutions	*Z_i_*	Similarity between each solution and the ideal one
*Z*(*n*_v_)	Importance degree of virtual node *n*_v_	*Z*(*n*_s_)	Importance degree of substrate node *n*_s_
*Can*(*n*_v*i*_)	Candidate substrate node set	*|N*_FN_|	Total number of faulty VNs

**Table 2:** Simulation parameters.

Characteristics	Values	Characteristics	Values
Number of substrate node	100	Number of virtual node per VN	[5, 10]
Substrate node capacity	[50, 100]	Virtual node capacity	[2, 10]
Number of substrate link	500	Virtual link connection probability	0.5
Substrate link capacity	[50, 100]	Virtual link capacity	[2, 10]
VN arrive rate	*Pois*{0.1}	VN lifetime	*Expo*{300}
Failure arrive rate	*Pois*{0.2}	Failure lifetime	*Expo*{500}
Network distance scope	1000 × 1000	Virtual node position constrain	400

**Table 3:** Comparison of faulty VN ranking methods.

Algorithm	Description
DVNRM-HMF-WRE	VN reconfiguration method proposed in this paper which ranks faulty VNs based on FVNRRM-WRE.
DVNRM-HMF-FN	VN reconfiguration method which ranks faulty VNs based on faulty component number.
DVNRM-HMF-VNR	VN reconfiguration method which ranks faulty VNs based on VN revenue.
DVNRM-HMF-RLT	VN reconfiguration method which ranks faulty VNs based on remaining lifetime of the faulty VN.

**Table 4:** Comparison of failure handling methods.

Algorithm	Description
DVNRM-HMF-WRE	VN reconfiguration method proposed in this paper which handles different failures based on HMFRA.
DVNRM-HMF-WRE II	VN reconfiguration method which handles VN link failure II first.
DVNRM-HMF-WRE III	VN reconfiguration method which handles VN link failure III first.

**Table 5:** Comparison of VN node migration methods.

Algorithm	Description
DVNRM-HMF-WRE	VN reconfiguration method proposed in this paper, in which virtual node is migrated based on VNMM-WRE.
DVNRM-HMF-RES	VN reconfiguration method in which virtual node is migrated based on the total node and link resources.
DVNRM-HMF-HOPS	VN reconfiguration method in which virtual node is migrated based on the reciprocal of hop counts between candidate substrate node and substrate node which is embedded by the neighboring virtual node of the migrated virtual node.

## Supplementary Materials

supplementary materials are available online at http://www.mdpi.com/1099-4300/20/9/711/s1.

## Abbreviations

The following abbreviations are used in this manuscript:

NV	network virtualization
VN	virtual network
SN	substrate network
SDN	software defined network
ILP	integer linear programming
DVNRM-HMF-WRE	dynamic virtual network reconfiguration method for hybrid multiple failures based on weighted relative entropy
FVNRRM-WRE	faulty VN reconfiguration ranking method based on weighted relative entropy
HMFRA	hybrid multiple failures ranking algorithm
VNMM-WRE	virtual node migration method based on weighted relative entropy
WRE	weighted relative entropy
AR	acceptance ratio
NOR	normal operation ratio
LAR/CR	long-term average revenue to cost ratio

## References

[1] Fischer A., Botero J.F., Beck M.T., de Meer H., Hesselbach X. (2013). Virtual network embedding: A survey. IEEE Commun. Surv. Tutor..

[2] Zhang J., Zhao C., Wu H., Lin M., Duan R. (2016). Virtual network embedding based on graph entropy. Entropy.

[3] Mijumbi R., Serrat J., Gorricho J.L., Bouten N., Turck F.D., Boutaba R. (2016). Network function virtualization: State-of-the-art and research challenges. IEEE Commun. Surv. Tutor..

[4] Tajiki M.M., Shojafar M., Akbari B., Salsano S., Conti M., Singhal M. Joint failure recovery, fault prevention, and energy-efficient resource management for real-time SFC in fog-supported SDN. Comput. Netw..

[5] Shojafar M., Canali C., Lancellotti R., Baccarelli E. Minimizing computing-plus-communication energy consumptions in virtualized networked data centers. Proceedings of the IEEE Symposium on Computers and Communication (ISCC).

[6] Shahriar N., Chowdhury S.R., Ahmed R. (2018). Virtual network survivability through joint space capacity allocation and embedding. IEEE J. Sel. Areas Commun..

[7] Boem F., Ferrari R.M.G., Keliris C., Parisini T., Polycarpou M. (2016). A distributed networked approach for fault detection of large-scale systems. IEEE Trans. Autom. Control.

[8] Herker S., Khan A., An X. Survey on survivable virtual network embedding problem and solutions. Proceedings of the 9th International Conference on Networking and Services (ICNS).

[9] Li R., Wu Q., Tan Y., Zhang J. (2018). On the optimal approach of survivable virtual network embedding in virtualized SDN. IEICE Trans. Inf. Syst..

[10] Sangjin H., Jason P.J., Pyungkoo P., Hosun Y., Hoyong R., Sungback R. (2016). Survivable virtual topology design in multi-domain optical networks. J. Opt. Commun. Netw..

[11] Aguado A., Davis M., Peng S., Alvarez M.V., Lopez V., Szyrkowiec T., Autenrieth A., Vilalta R., Mayoral A., Muñoz R. (2016). Dynamic virtual network reconfiguration over SDN orchestrated multi-technology optical transport domains. J. Lightw. Technol..

[12] Chowdhury S.R., Ahmed R., Khan M.M.A., Shahriar N., Boutaba R., Mitra J., Zeng F. (2016). Dedicated protection for survivable virtual network embedding. IEEE Trans. Netw. Serv. Manag..

[13] Ayoubi S., Chen Y., Assi C. (2016). Towards promoting backup-sharing in survivable virtual network design. IEEE/ACM Trans. Netw..

[14] Chang X., Muppala J.K., Wang B., Liu J., Sun L. Migration cost aware virtual network re-embedding in presence of resource failures. Proceedings of the 18th IEEE International Conference on Networks (ICON).

[15] Peng L. (2015). A topology-awareness virtual network reconfiguration algorithm. J. Sichuan Univ..

[16] Ghaleb A.M., Khalifa T., Ayoubi S., Shaban K.B. Surviving link failures in multicast VN embedded applications. Proceedings of the IEEE/IFIP Network Operations and Management Symposium.

[17] Qiang Z., Qiang W., Sheng F., Wu L. Heuristic survivable virtual network embedding based on node migration and link remapping. Proceedings of the IEEE 7th Joint International Information Technology and Artificial Intelligence Conference.

[18] Shahriar N., Ahmed R., Khan A., Chowdhury S.R., Boutaba R., Mitra J., David R. ReNoVatE: Recovery from node failure in virtual network embedding. Proceedings of the 12th International Conference on Network and Service Management (CNSM).

[19] Shahriar N., Ahmed R., Chowdhury S.R., Khan A., Boutaba R., Mitra J. (2017). Generalized recovery from node failure in virtual network embedding. IEEE Trans. Netw. Serv. Manag..

[20] Gong S., Chen J., Zhao S., Zhu Q. (2016). Virtual network embedding with multi-attribute node ranking based on TOPSIS. KSII Trans. Int. Inf. Syst..

[21] Su Y., Meng X., Meng Q., Zhao Z. (2018). Environment adaptive and joint topology aware virtual network embedding algorithm. J. Electr. Inf. Technol..

[22] Zhu Y., Ammar M. Algorithms for assigning substrate network resources to virtual network components. Proceedings of the 25th IEEE International Conference on Computer Communications.

[23] Gong S., Chen J., Huang C., Zhu Q. (2015). Trust-aware secure virtual network embedding algorithm. J. Commun..

[24] Zhao Z., Meng X., Su Y., Li Z. (2017). Virtual network embedding based on node connectivity awareness and path integration evaluation. KSII Trans. Int. Inf. Syst..

