# Supplementary figures and images for: Dynamic Virtual Network Reconfiguration Method for Hybrid Multiple Failures Based on Weighted Relative Entropy
Source: Entropy (Basel). 2018 Sep 15;20(9):711. doi: 10.3390/e20090711 (PMC7513238; doi:10.3390/e20090711)

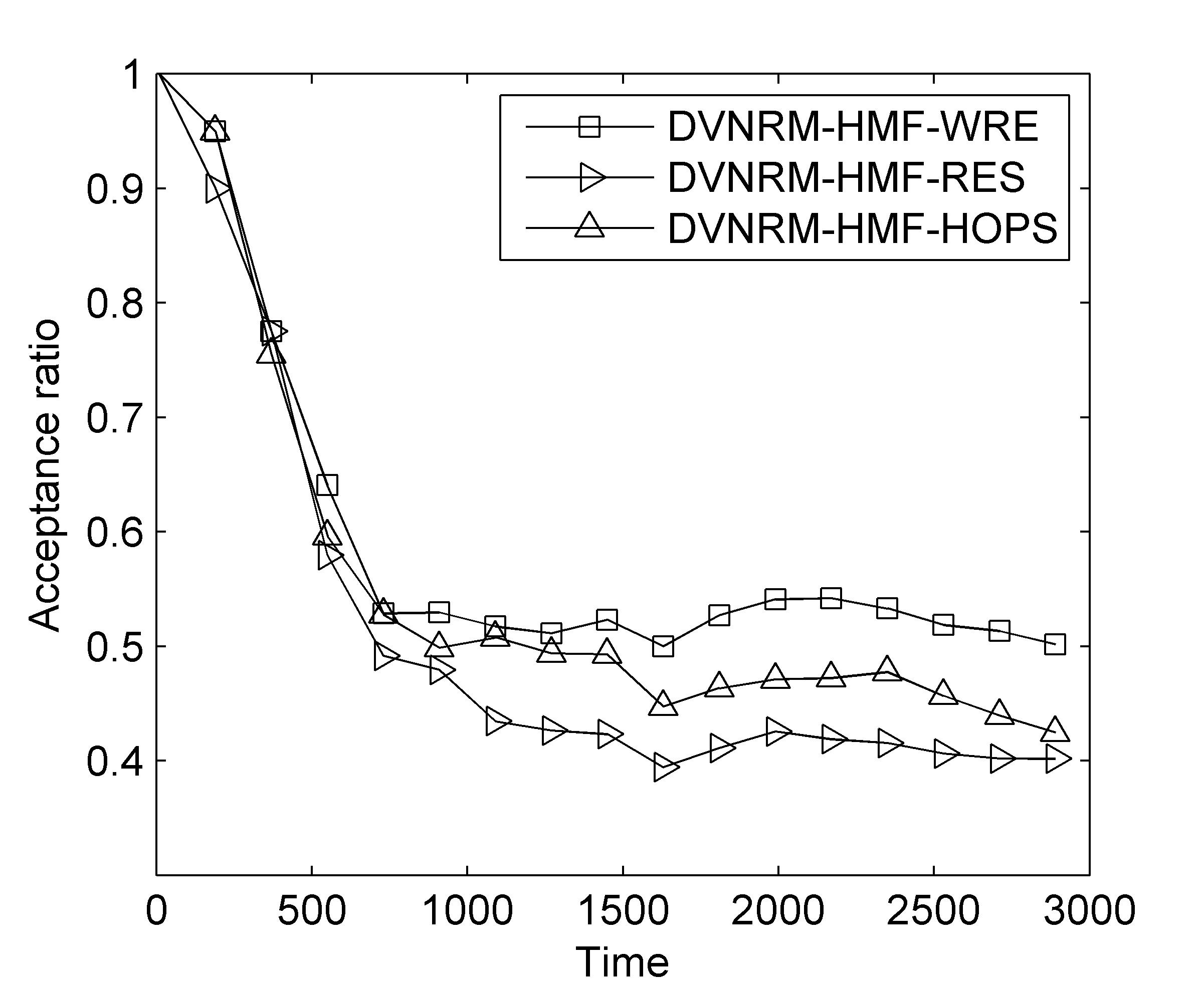

Supplement: Supplementary file 1 [file entropy-20-00711-s001.zip › supplementary files/final resaults/Comparison of different VN node migration methods/FIGURE S6(a).jpg]

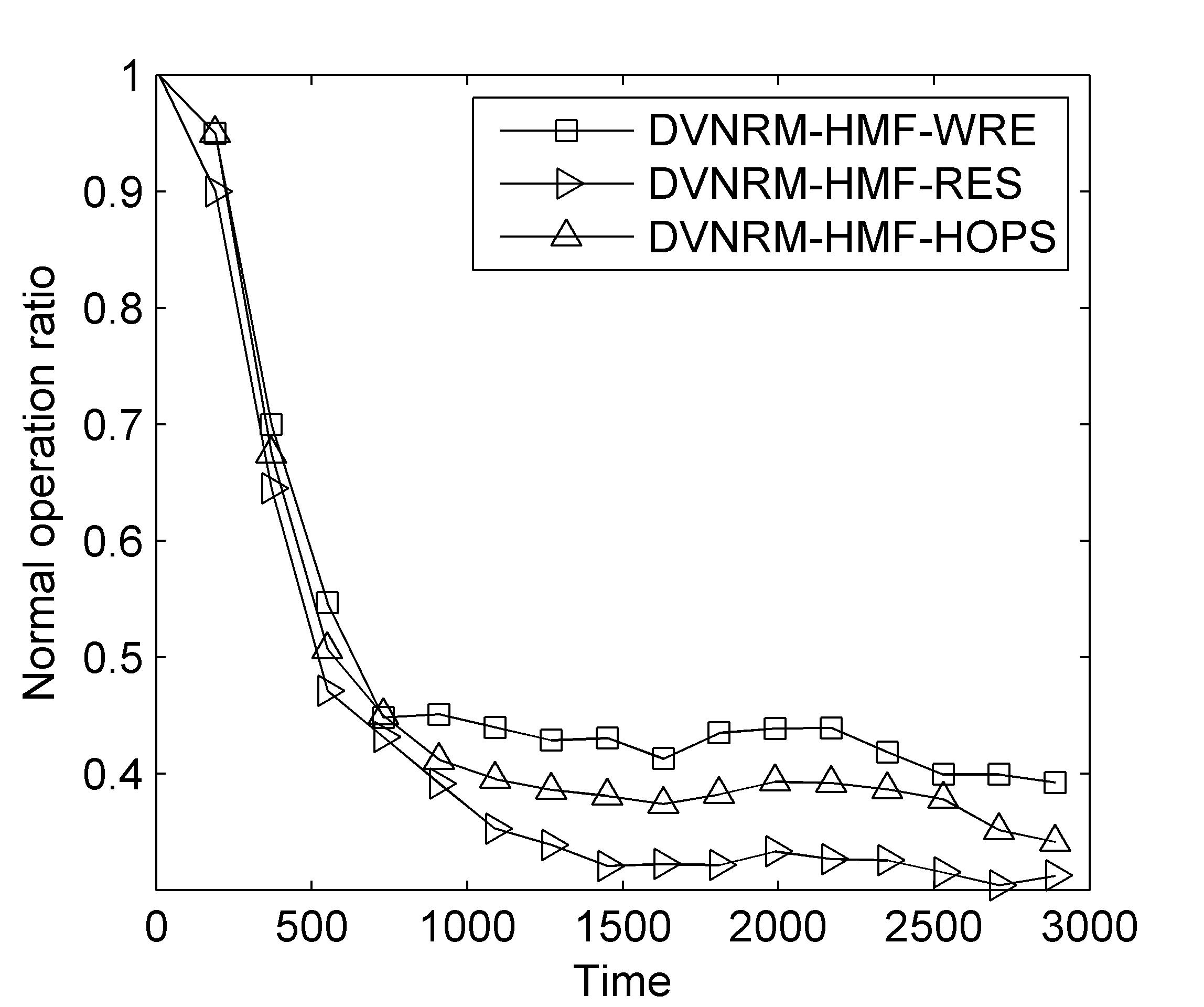

Supplement: Supplementary file 1 [file entropy-20-00711-s001.zip › supplementary files/final resaults/Comparison of different VN node migration methods/FIGURE S6(b).jpg]

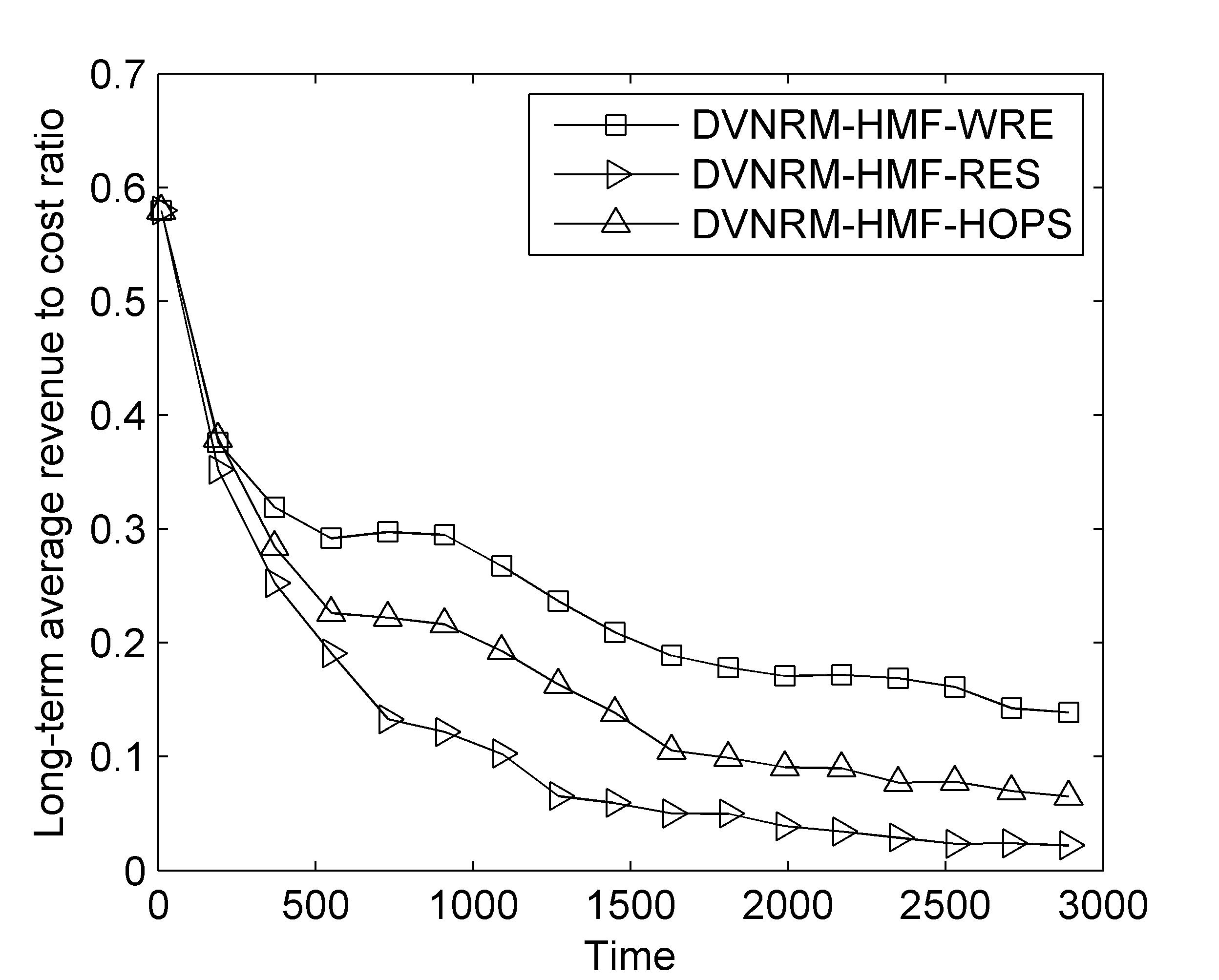

Supplement: Supplementary file 1 [file entropy-20-00711-s001.zip › supplementary files/final resaults/Comparison of different VN node migration methods/FIGURE S6(c).jpg]

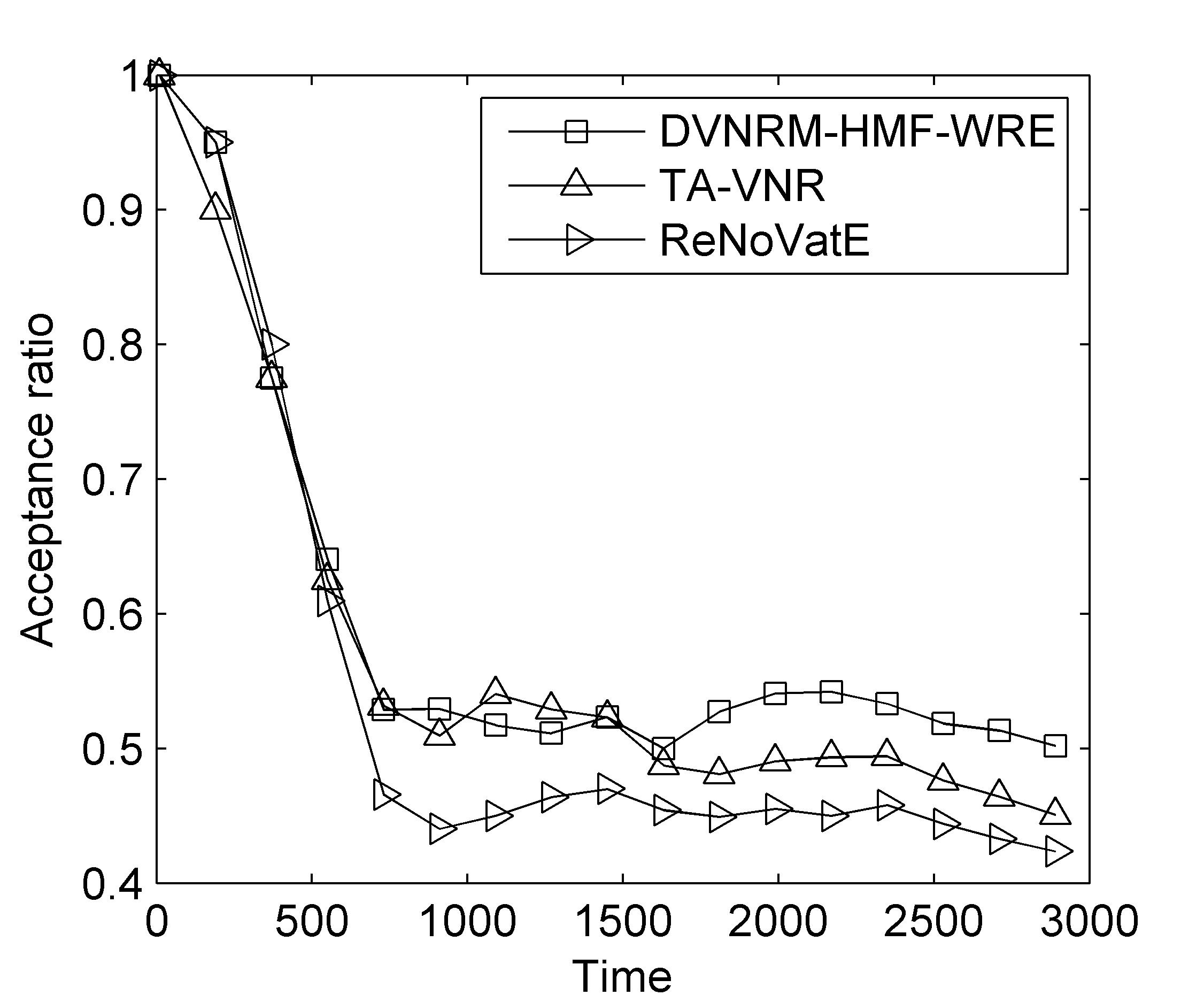

Supplement: Supplementary file 1 [file entropy-20-00711-s001.zip › supplementary files/final resaults/Comparison of different VN reconfiguration methods/FIGURE S3(a).jpg]

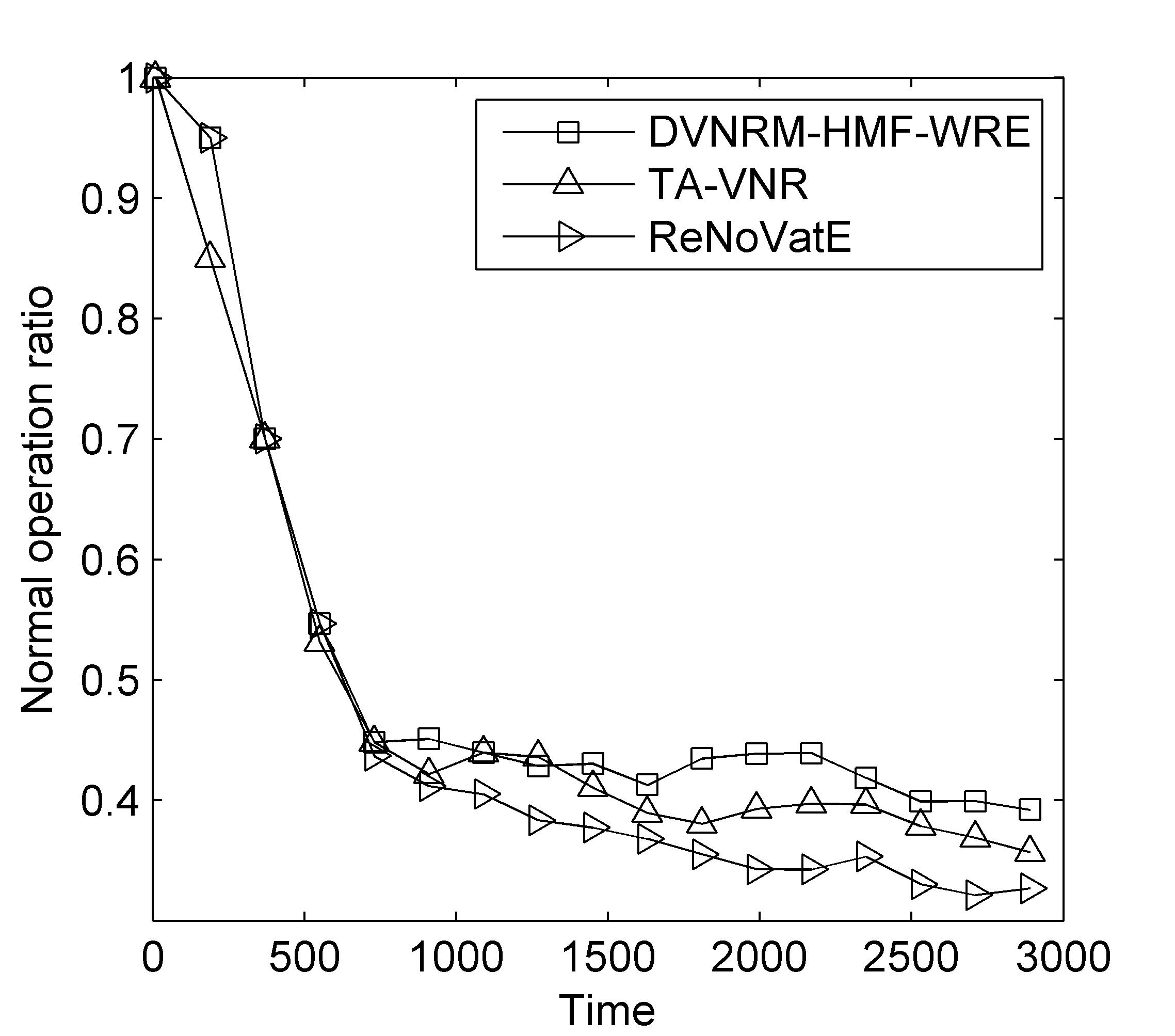

Supplement: Supplementary file 1 [file entropy-20-00711-s001.zip › supplementary files/final resaults/Comparison of different VN reconfiguration methods/FIGURE S3(b).jpg]

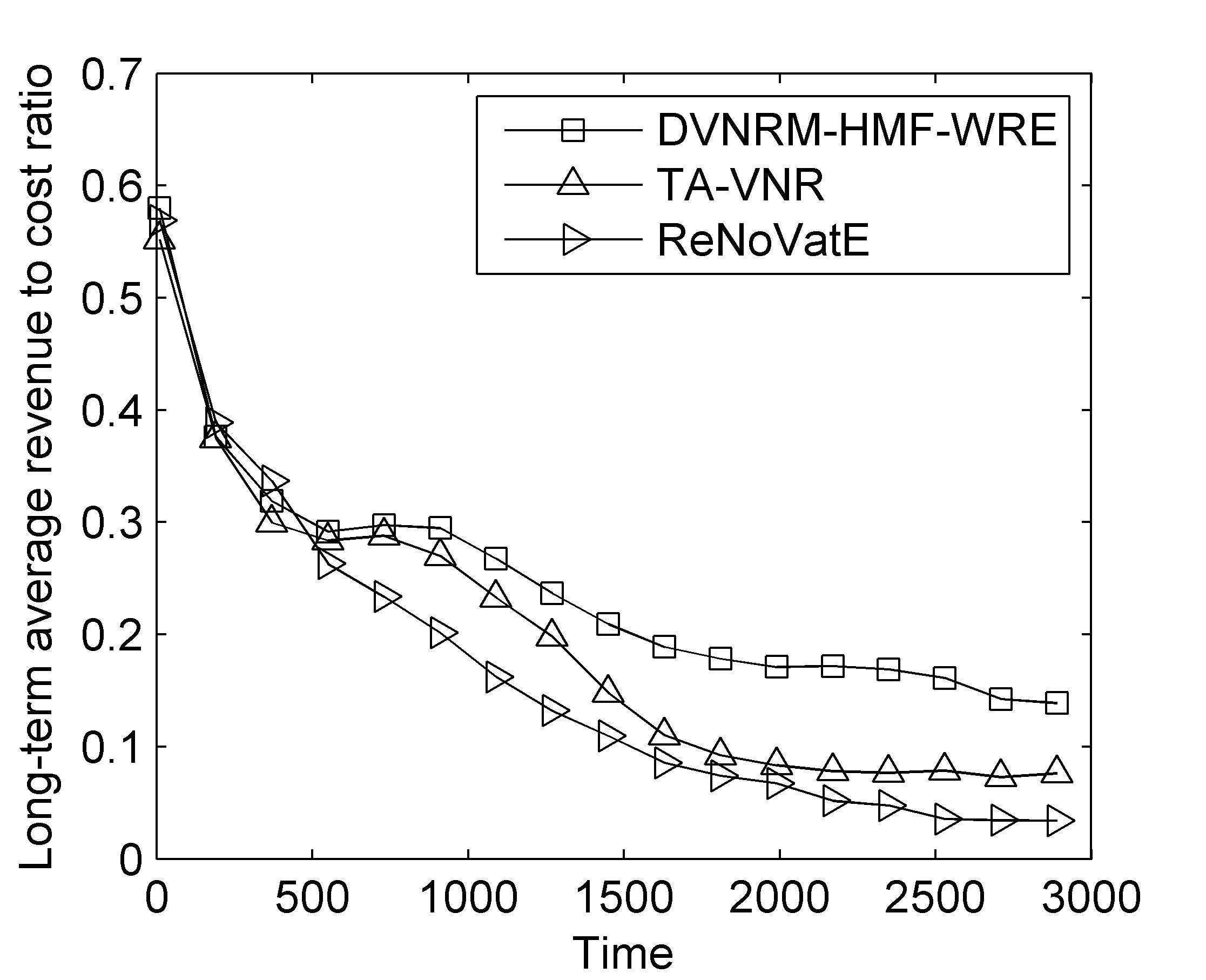

Supplement: Supplementary file 1 [file entropy-20-00711-s001.zip › supplementary files/final resaults/Comparison of different VN reconfiguration methods/FIGURE S3(c).jpg]

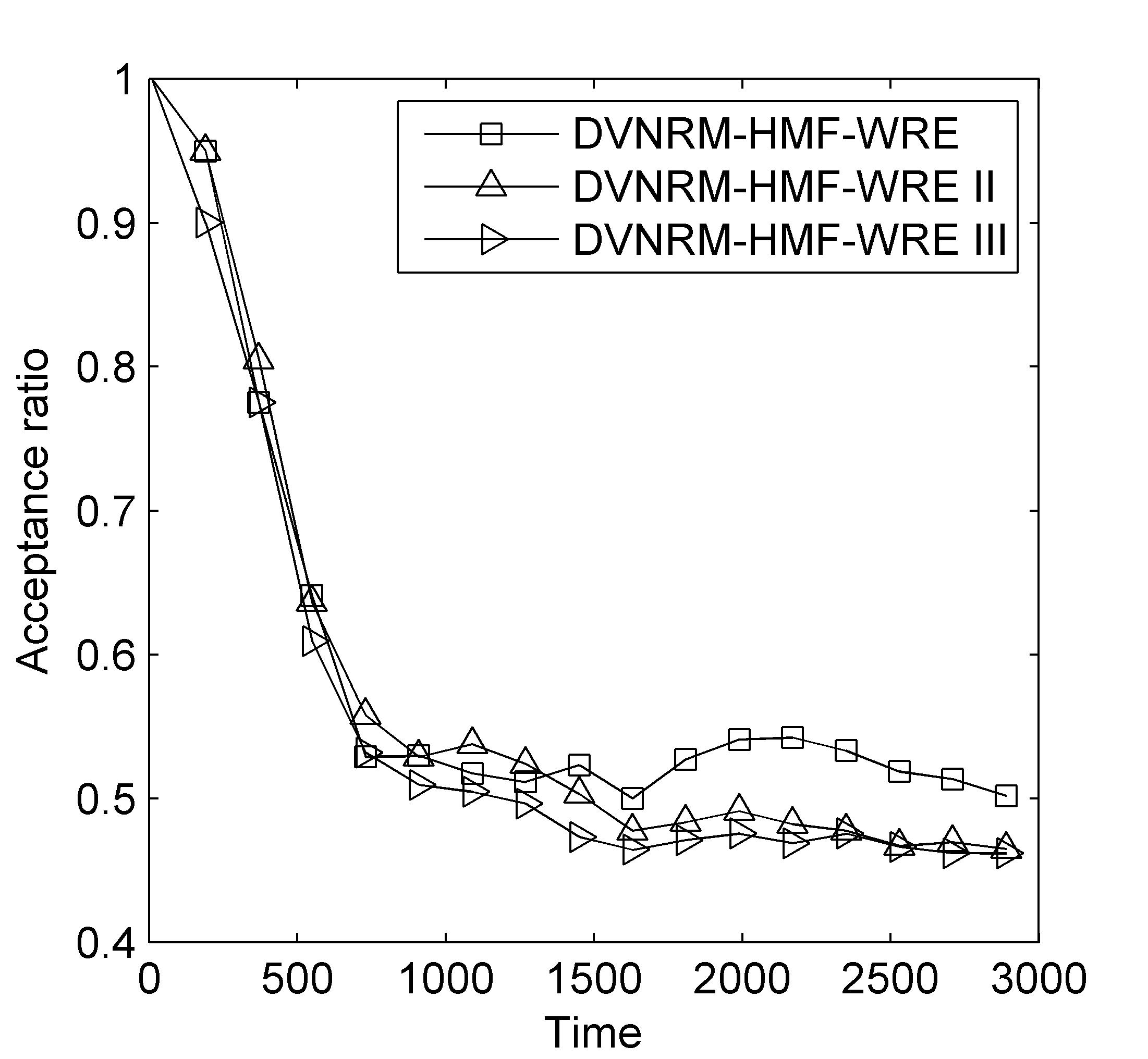

Supplement: Supplementary file 1 [file entropy-20-00711-s001.zip › supplementary files/final resaults/Comparison of failure handling methods/FIGURE S5(a).jpg]

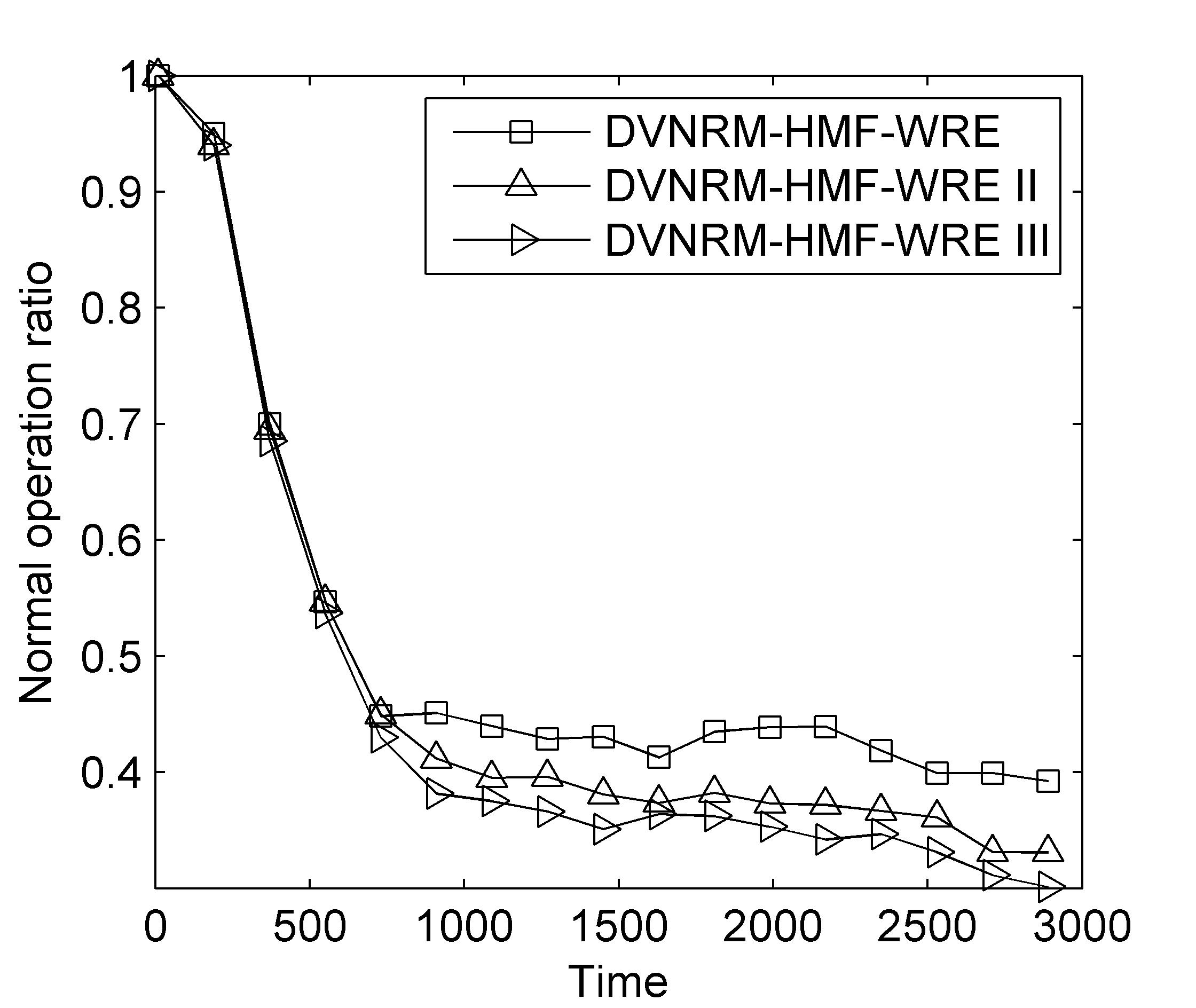

Supplement: Supplementary file 1 [file entropy-20-00711-s001.zip › supplementary files/final resaults/Comparison of failure handling methods/FIGURE S5(b).jpg]

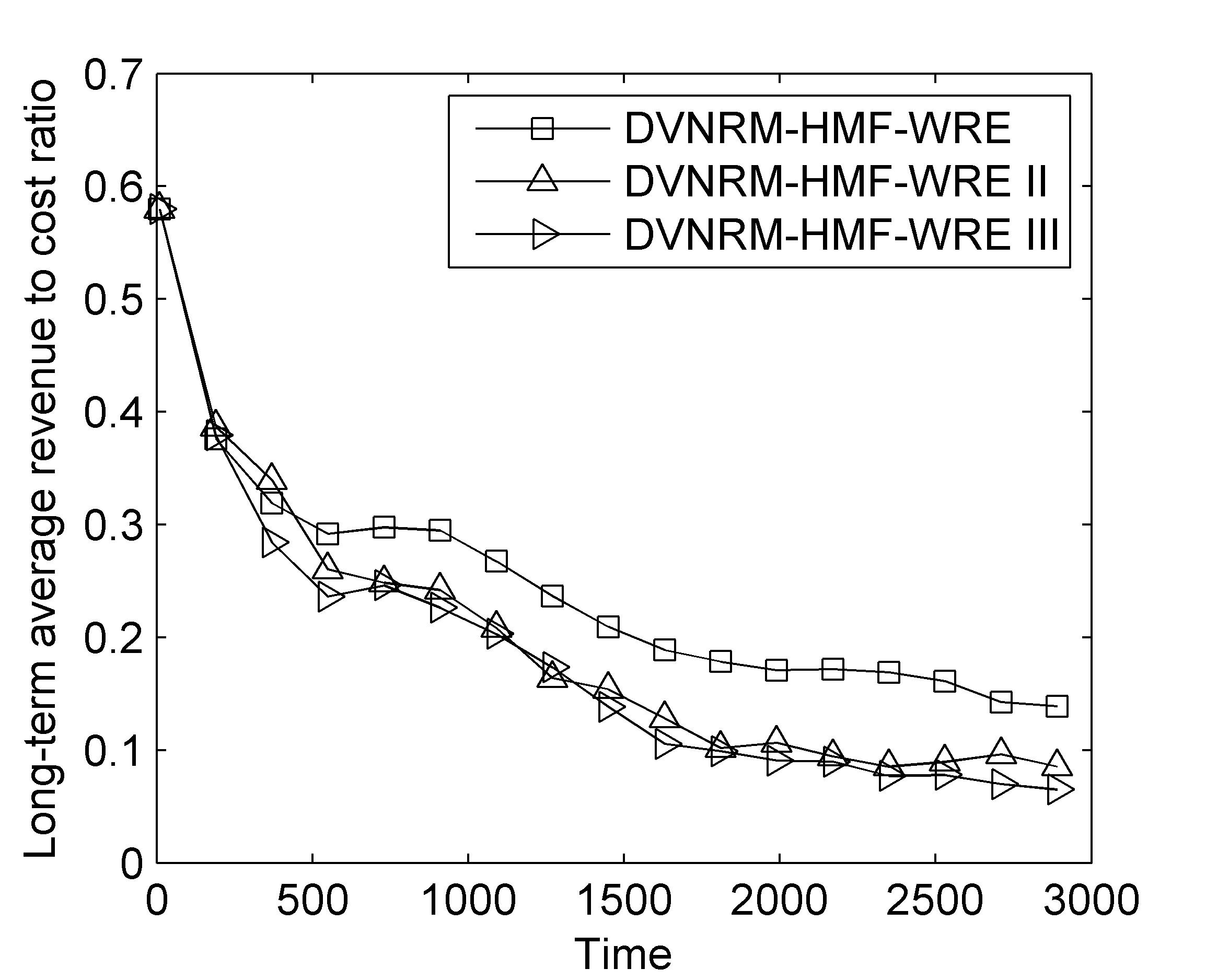

Supplement: Supplementary file 1 [file entropy-20-00711-s001.zip › supplementary files/final resaults/Comparison of failure handling methods/FIGURE S5(c).jpg]

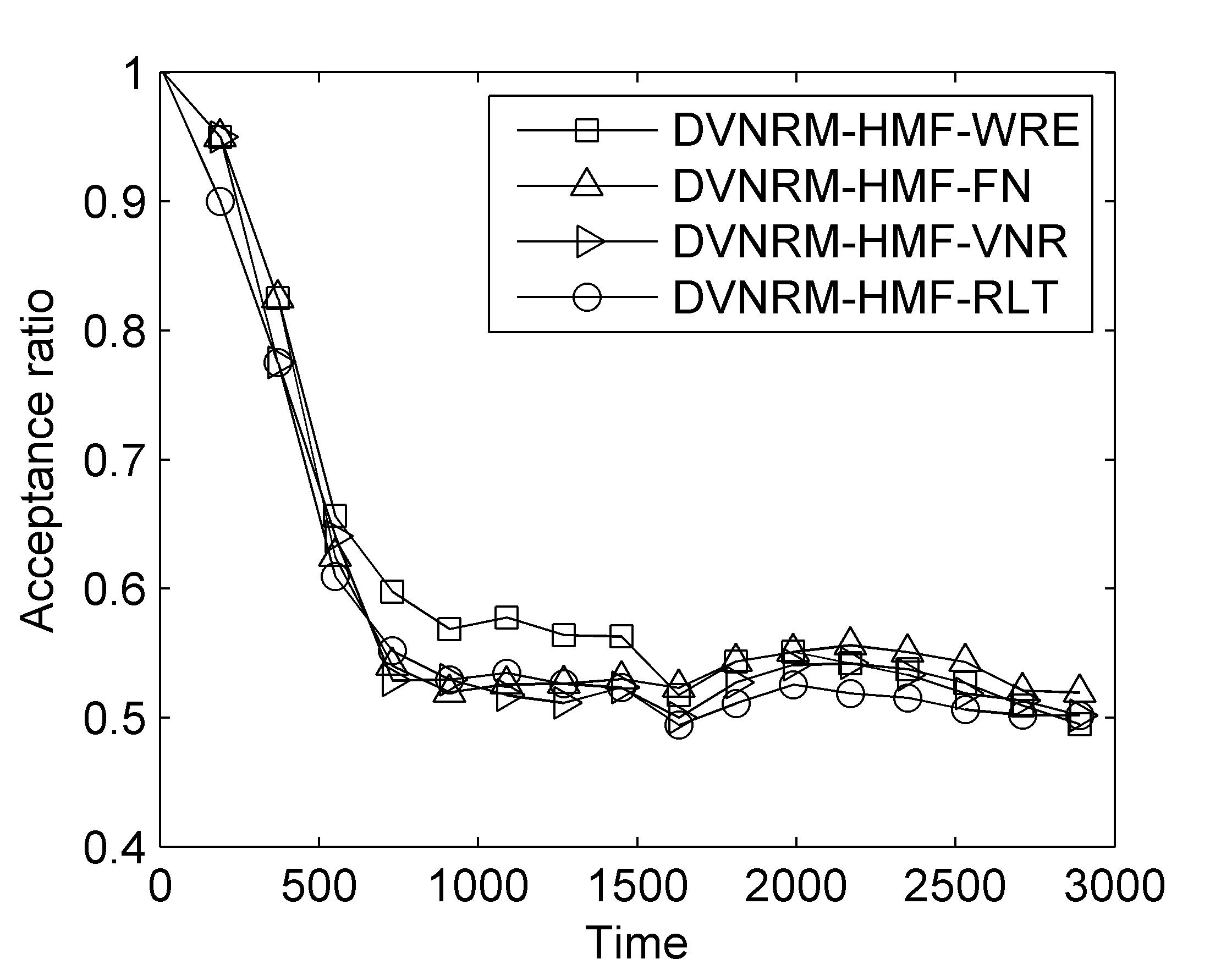

Supplement: Supplementary file 1 [file entropy-20-00711-s001.zip › supplementary files/final resaults/Comparison of faulty VN ranking methods/FIGURE S4(a).jpg]

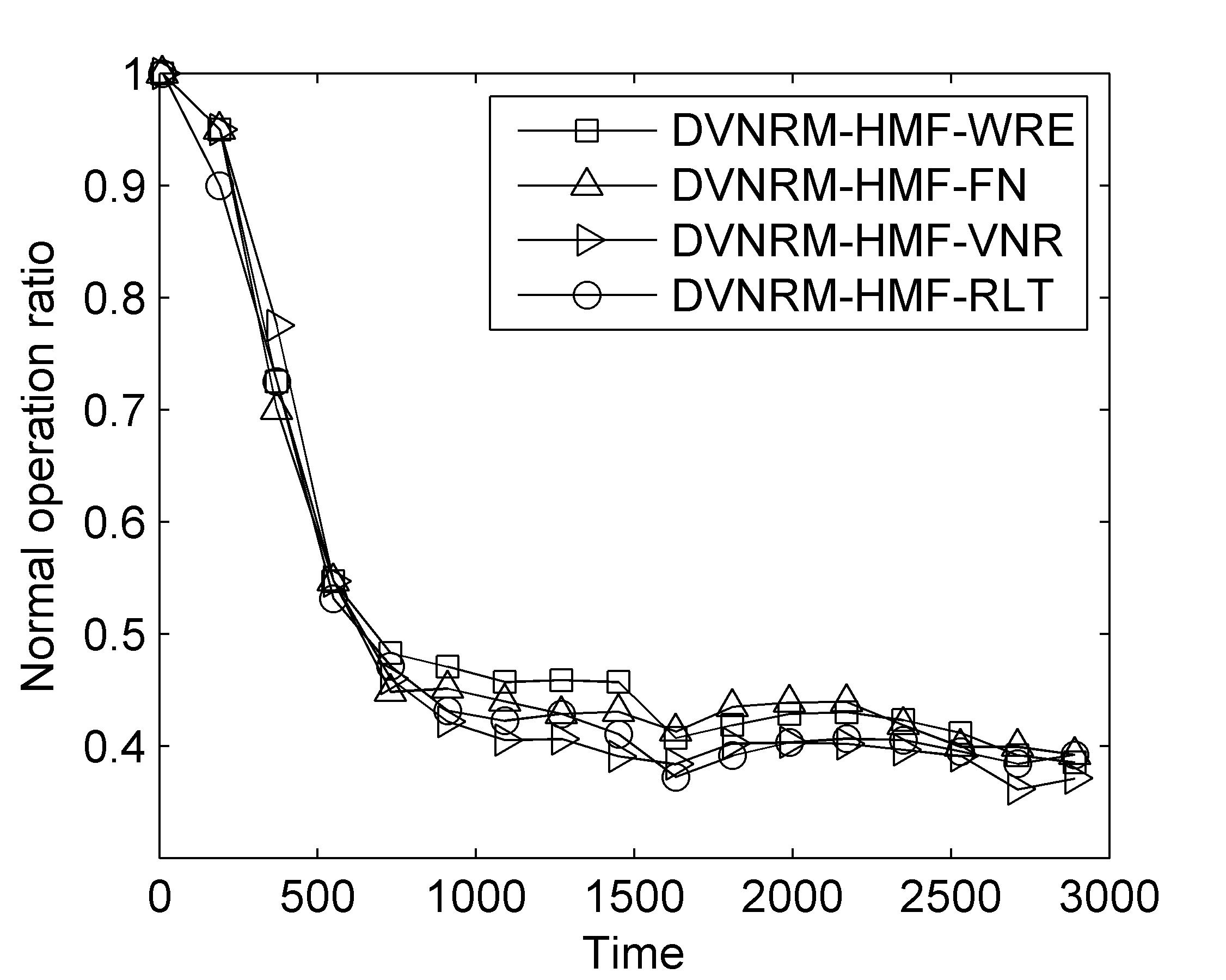

Supplement: Supplementary file 1 [file entropy-20-00711-s001.zip › supplementary files/final resaults/Comparison of faulty VN ranking methods/FIGURE S4(b).jpg]

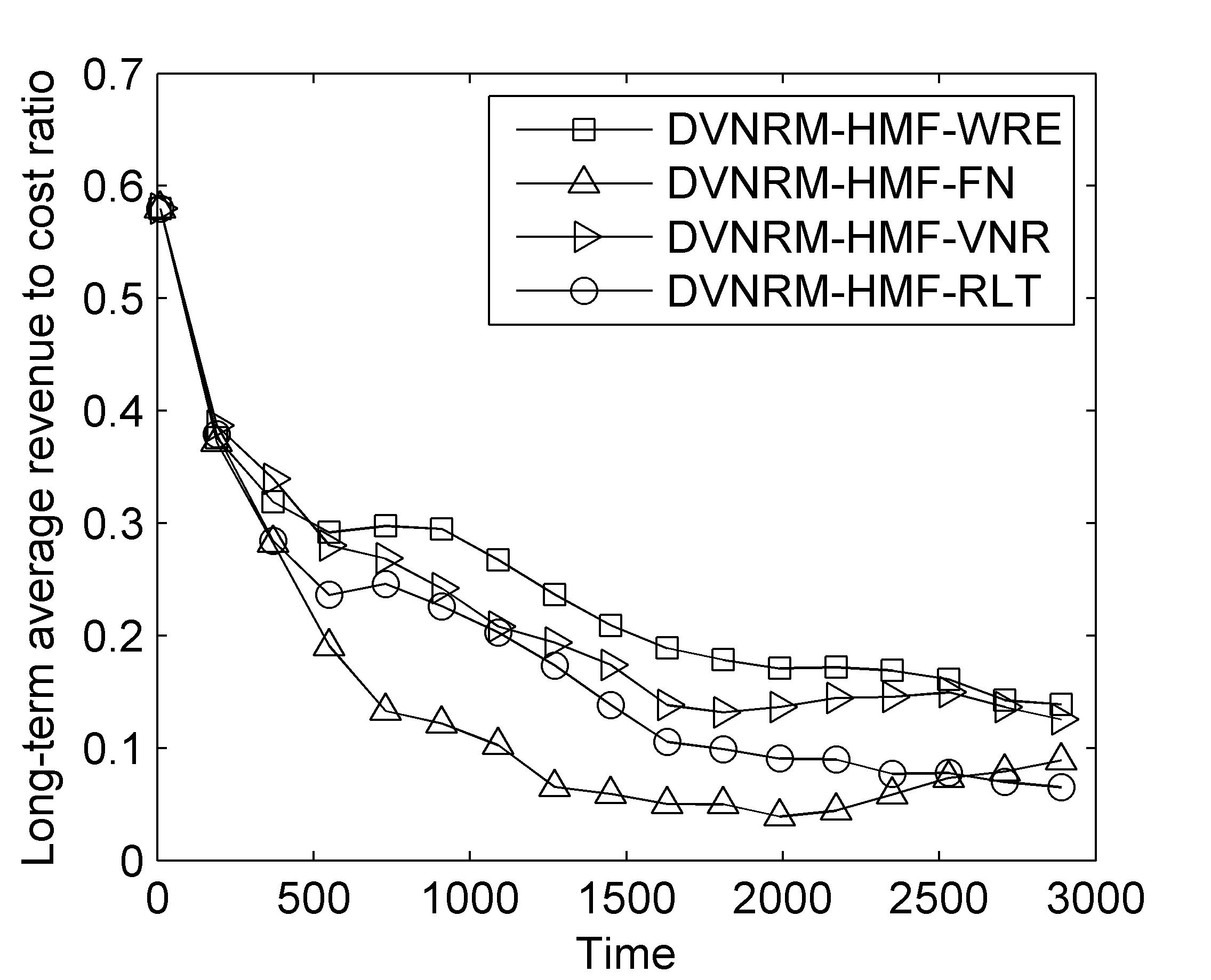

Supplement: Supplementary file 1 [file entropy-20-00711-s001.zip › supplementary files/final resaults/Comparison of faulty VN ranking methods/FIGURE S4(c).jpg]

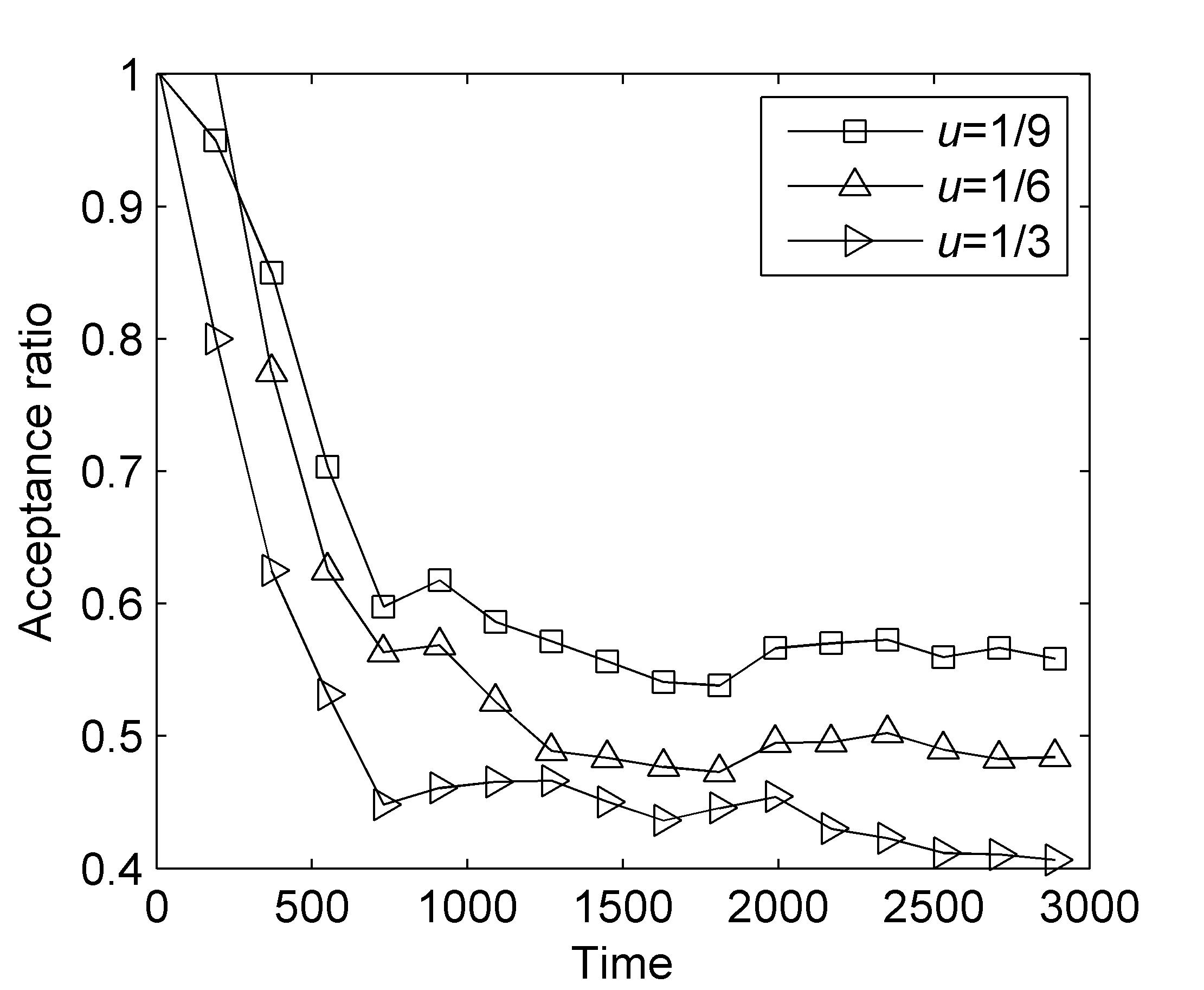

Supplement: Supplementary file 1 [file entropy-20-00711-s001.zip › supplementary files/final resaults/The arrival rate of failure u/FIGURE S7(a).jpg]

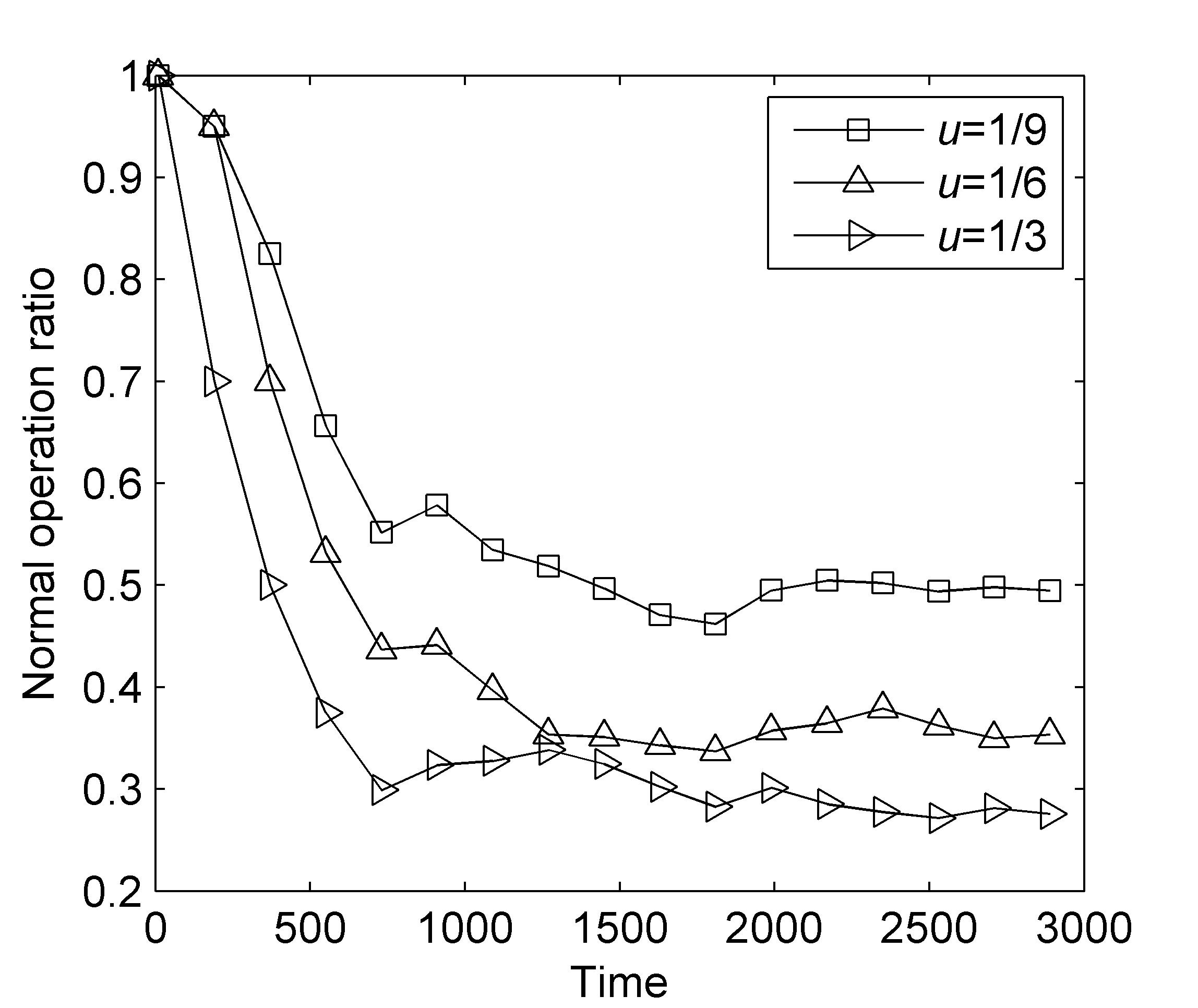

Supplement: Supplementary file 1 [file entropy-20-00711-s001.zip › supplementary files/final resaults/The arrival rate of failure u/FIGURE S7(b).jpg]

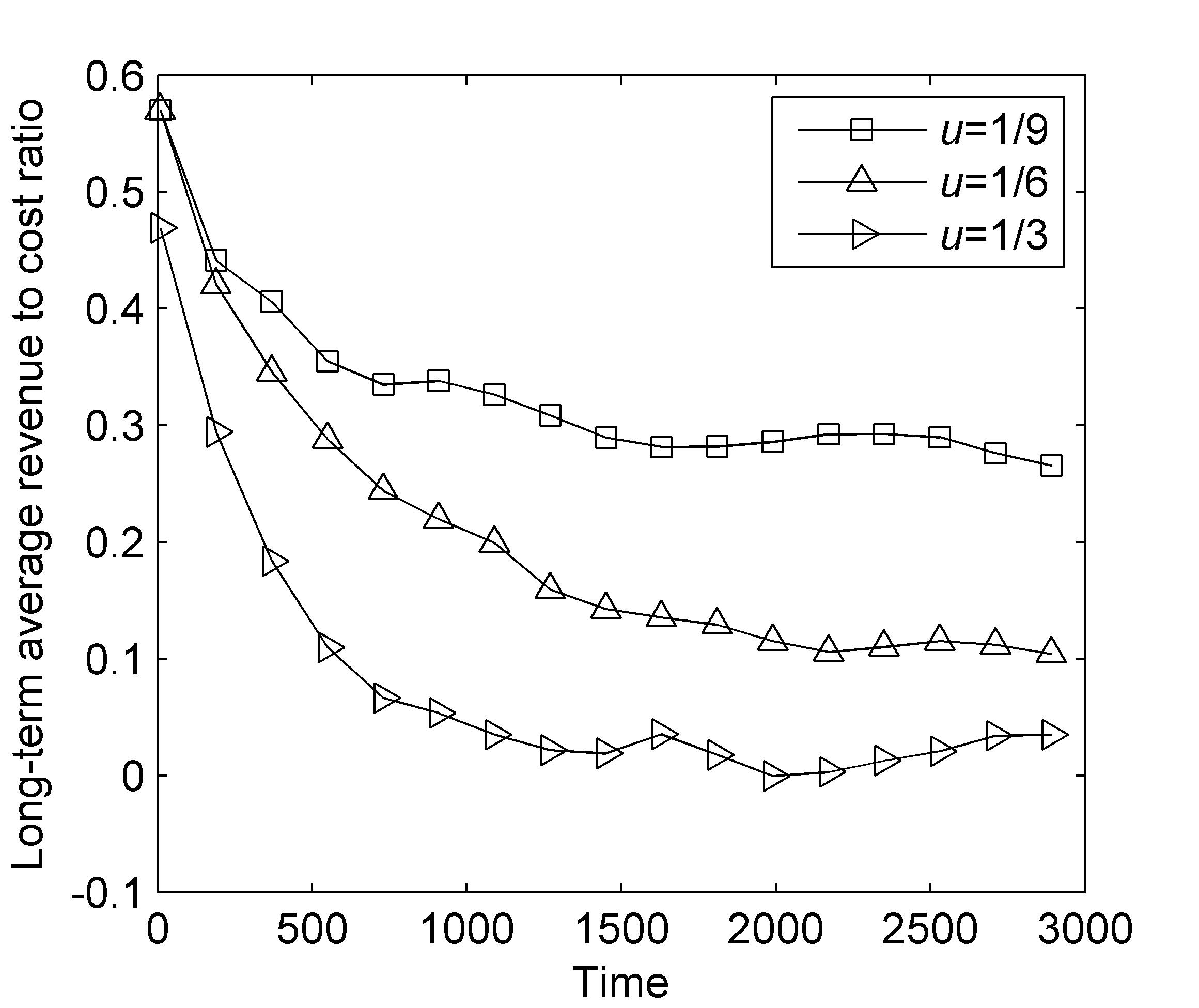

Supplement: Supplementary file 1 [file entropy-20-00711-s001.zip › supplementary files/final resaults/The arrival rate of failure u/FIGURE S7(c).jpg]

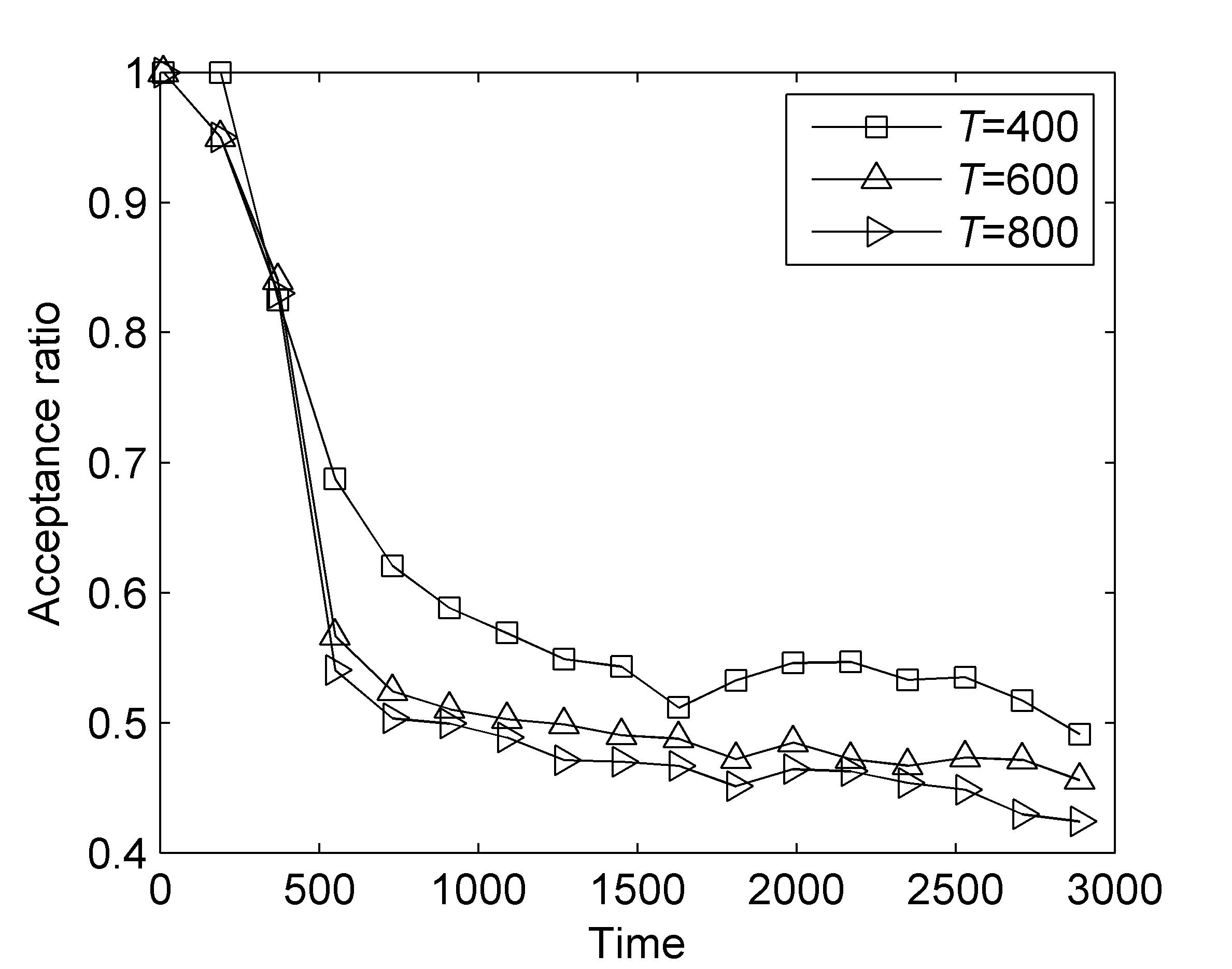

Supplement: Supplementary file 1 [file entropy-20-00711-s001.zip › supplementary files/final resaults/The lifetime of failure T/FIGURE S8(a).jpg]

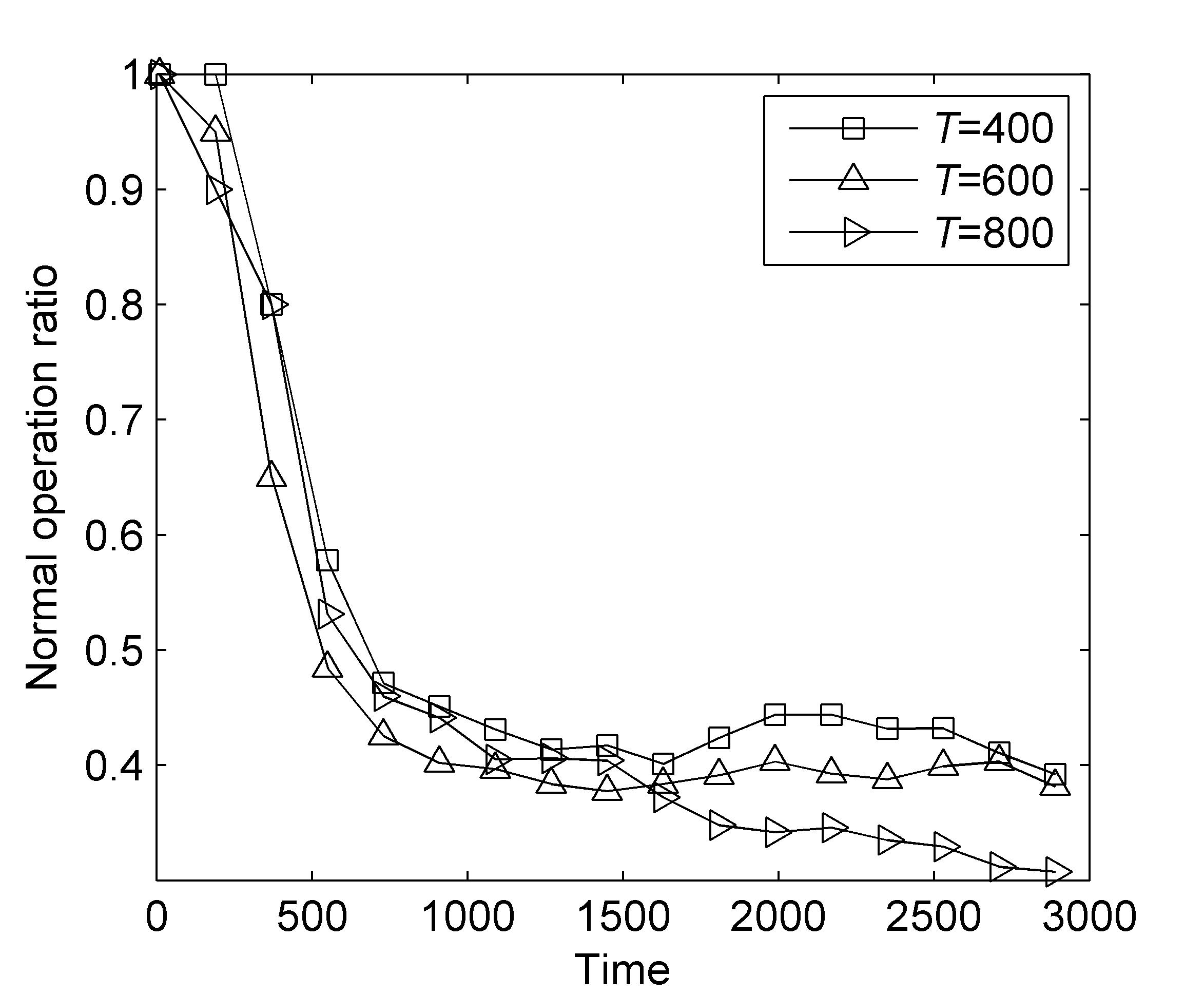

Supplement: Supplementary file 1 [file entropy-20-00711-s001.zip › supplementary files/final resaults/The lifetime of failure T/FIGURE S8(b).jpg]

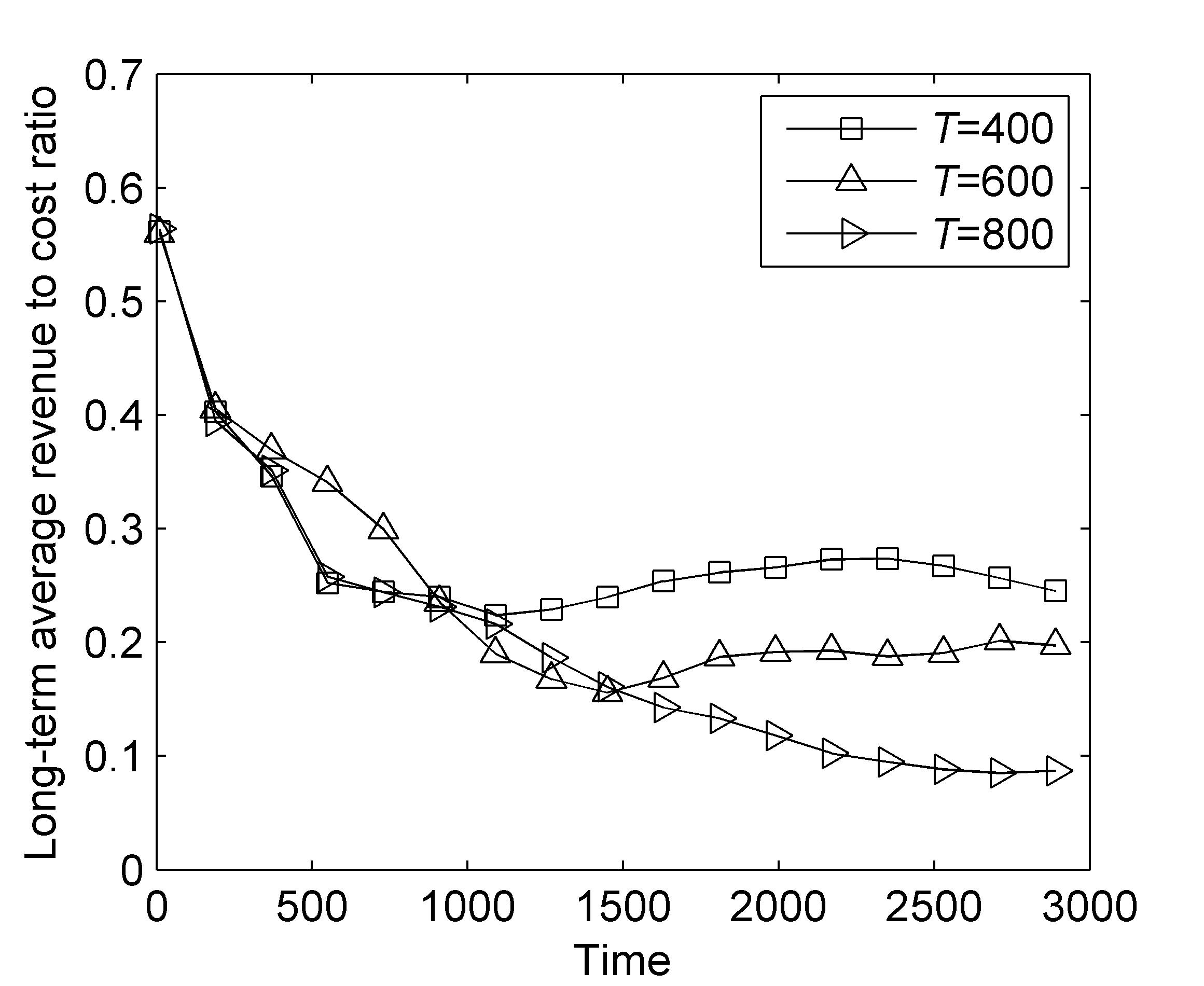

Supplement: Supplementary file 1 [file entropy-20-00711-s001.zip › supplementary files/final resaults/The lifetime of failure T/FIGURE S8(c).jpg]
